# Serious games for upper limb rehabilitation after stroke: a meta-analysis

**DOI:** 10.1186/s12984-021-00889-1

**Published:** 2021-06-15

**Authors:** Ioannis Doumas, Gauthier Everard, Stéphanie Dehem, Thierry Lejeune

**Affiliations:** 1 Institut de Recherche Expérimentale et Clinique, Neuro Musculo Skeletal Lab (NMSK), Secteur des Sciences de la Santé, Université Catholique de Louvain, Avenue Mounier 53, 1200 Brussels, Belgium; 2 Service de Médecine Physique et Réadaptation, Cliniques Universitaires Saint-Luc, Avenue Hippocrate 10, 1200 Brussels, Belgium; 3 Université Catholique de Louvain, Louvain Bionics, 1348 Louvain-la-Neuve, Belgium

**Keywords:** Stroke, Upper extremity, Serious games, Virtual reality, Robotics

## Abstract

**Background:**

Approximately two thirds of stroke survivors maintain upper limb (UL) impairments and few among them attain complete UL recovery 6 months after stroke. Technological progress and gamification of interventions aim for better outcomes and constitute opportunities in self- and tele-rehabilitation.

**Objectives:**

Our objective was to assess the efficacy of serious games, implemented on diverse technological systems, targeting UL recovery after stroke. In addition, we investigated whether adherence to neurorehabilitation principles influenced efficacy of games specifically designed for rehabilitation, regardless of the device used.

**Method:**

This systematic review was conducted according to PRISMA guidelines (PROSPERO registration number: 156589). Two independent reviewers searched PubMed, EMBASE, SCOPUS and Cochrane Central Register of Controlled Trials for eligible randomized controlled trials (PEDro score ≥ 5). Meta-analysis, using a random effects model, was performed to compare effects of interventions using serious games, to conventional treatment, for UL rehabilitation in adult stroke patients. In addition, we conducted subgroup analysis, according to adherence of included studies to a consolidated set of 11 neurorehabilitation principles.

**Results:**

Meta-analysis of 42 trials, including 1760 participants, showed better improvements in favor of interventions using serious games when compared to conventional therapies, regarding UL function (SMD = 0.47; 95% CI = 0.24 to 0.70; *P* < 0.0001), activity (SMD = 0.25; 95% CI = 0.05 to 0.46; *P* = 0.02) and participation (SMD = 0.66; 95% CI = 0.29 to 1.03; *P* = 0.0005). Additionally, long term effect retention was observed for UL function (SMD = 0.42; 95% CI = 0.05 to 0.79; *P* = 0.03). Interventions using serious games that complied with at least 8 neurorehabilitation principles showed better overall effects. Although heterogeneity levels remained moderate, results were little affected by changes in methods or outliers indicating robustness.

**Conclusion:**

This meta-analysis showed that rehabilitation through serious games, targeting UL recovery after stroke, leads to better improvements, compared to conventional treatment, in three ICF-WHO components. Irrespective of the technological device used, higher adherence to a consolidated set of neurorehabilitation principles enhances efficacy of serious games. Future development of stroke-specific rehabilitation interventions should further take into consideration the consolidated set of neurorehabilitation principles.

**Supplementary Information:**

The online version contains supplementary material available at 10.1186/s12984-021-00889-1.

## Background

Each year more than 1 million Europeans suffer from stroke and approximately two-thirds of survivors maintain upper limb (UL) paresis [1]. This number is expected to rise by 35% in upcoming years [2] leading to additional rehabilitation needs. To this date, few people attain complete UL recovery 6 months after stroke [3]. New interventions targeting the UL aim for better outcomes in activities of daily living (ADL), functional independence and quality of life. Alongside conventional therapies, recent developments offer possibilities in self- and tele-rehabilitation [4] which could help manage, cost-efficiently [5], increasing rehabilitation demands.

Technological improvements in robot assisted therapy (RAT) and virtual reality (VR) systems (VRS) enhance patient care and facilitate therapist assistance during UL rehabilitation [6, 7]. First, RAT promotes the use of the affected limb, intensifies rehabilitation through task repetition and offers task-specific practice [7]. Effectiveness of RAT is established for UL rehabilitation after stroke [8, 9]. Secondly, VRS provide augmented feedback, preserve motivation and are becoming cost-efficient [5]. Recent meta-analyses suggest a superior effect of VR-based interventions compared to conventional treatment on UL function and activity after stroke, especially if developed for this specific purpose [10–12]. Authors attributed these findings to the fact that VRS specifically developed for rehabilitation, as opposed to commercial video-games (CVG), fulfil numerous neurorehabilitation principles.

Typically, a common denominator of VRS and RAT is playful interventions by means of serious games [13, 14]. A serious game is defined as a game that has education or rehabilitation as primary goal. These games combine entertainment, attentional engagement and problem solving to challenge function and performance [15, 16]. Moreover, they comply with several motor relearning principles that constitute the basis of effective interventions in neurorehabilitation [11, 16]. For example, some devices adapt game difficulty to stimulate recovery and maintain motivation [15]. Others incorporate functional tasks mimicking ADL in virtual environments and provide performance feedback during and/or after task completion [17]. Characteristics of serious games differ depending on targeted rehabilitation purposes and technical specificities of the system they are implemented on.

Previous work on the efficacy of VR-based interventions indicated that serious games may enhance UL recovery after stroke [11, 12, 18]. However, why such interventions, specifically developed for rehabilitation purposes and implemented on various types of devices (such as robots, smartphones, tablets, motion capture systems, etc.), may constitute effective therapies for UL rehabilitation after stroke needs to be further investigated. Recent theoretical research proposed consolidation of commonly acknowledged neurorehabilitation principles [16]. Usually, serious games comply with several of these principles which creates an opportunity to evaluate clinical applicability of the consolidated set of principles. To this day, it remains unclear whether higher adherence to this consolidated set of neurorehabilitation principles enhances efficacy of interventions. In addition, it is not well known whether adherence to specific principles influences efficacy. Finally, rehabilitation effects on participation outcomes remain relatively unexplored. In this context, efficacy of interventions should be addressed in terms of all components of the World Health Organization’s International Classification of Function, Disability, and Health (ICF-WHO) model [19].

The main objective of this systematic review and meta-analysis was to address the following question in PICOS form: in adults after stroke (P), do serious games, implemented on various technological systems (I), show better efficacy than conventional treatment (C), to rehabilitate UL function and activity, and patient’s participation (O)? A secondary objective was to assess whether higher adherence to a consolidated set of neurorehabilitation principles enhances efficacy of games specifically designed for rehabilitation, irrespective of the technological device used.

## Methods

### Design

This systematic review followed the Preferred Reporting Items for Systematic Reviews and Meta-Analysis (PRISMA) guidelines [20]. The protocol was registered in International Prospective Register of Systematic Reviews (PROSPERO 2020, registration number: 156589).

### Identification and study selection

A search strategy looking for relevant literature was developed for PubMed and adapted for the other databases, namely Scopus, Embase and Cochrane Library (Additional file 1). Authors received help from a professional librarian to set up the search strategy. Two investigators (GE and ID) independently retrieved studies. All references were stored in reference management software EndNote X9. After removal of duplicates, remaining references were first screened based on titles and abstracts.

Study eligibility was assessed according to the following criteria: (a) design of randomized controlled trials (RCT) (b) participants were adults undergoing stroke rehabilitation (c) the intervention consisted of games developed for neurorehabilitation purposes and implemented in the following technological devices: robotic systems, VRS, tablets, smartphones and motion capture systems (d) relevant outcomes were employed to assess UL function, UL activity and participation (e) studies were published in French or English before May 5th, 2020. All studies using additional therapeutic modalities such as brain stimulation, electrical stimulation or invasive treatments were excluded.

Systematic reviews assessing effectiveness of VR-based rehabilitation and RAT in stroke recovery were also hand-searched looking for relevant references. Finally, a selection based on full-text was conducted by the same two reviewers. Disagreements were resolved through discussion.

### Quality and risk of bias assessment

The PEDro checklist was used for methodological quality assessment of trials [21]. In addition, the Cochrane Collaboration’s Risk of Bias (RoB) tool was employed to conduct a critical appraisal of each trial’s internal validity [22].

### Data extraction

The following data concerning patients, interventions, control groups and outcomes were extracted from each study: number of patients enrolled in each group, mean time since stroke, corresponding stroke stage classification (subacute: 7 days to 6 months after stroke, chronic: more than 6 months after stroke) [23], dosage and duration of the intervention, technological device used, type and duration of treatment for the control group, presence of a follow-up assessment and outcomes assessed in each timepoint evaluation.

Studies were also assessed in terms of the number of neurorehabilitation principles their intervention fulfilled as described in the review of Maier et al. [11]. These authors described a total of 11 principles presented in Table 1. The two reviewers, independently, investigated whether interventions of included studies fulfilled each one of the neurorehabilitation principles. For each clearly identified principle, one point was attributed to the study. In case available information was vague, missing or did not match the neurorehabilitation principle descriptive’ (as mentioned in Table 1), no point was accorded. Then, we calculated a total score out of 11 for each included study.

**Table 1:** List of neurorehabilitation principles with description established by Maier et al. [11, 16]

Neurorehabilitation principle	Description	Fulfilled in studies (%)		
		All studies	+	=
Massed practice	Tasks aiming to increase the number of repetitions performed	81	79	85
Dosage	Intensive training: more than a daily session of 60 min on every weekday	52	59	38
Structured practice	Training that includes periods of rest	26	31	15
Task-specific practice	Functional training relevant to ADL	100	100	100
Variable practice	Training that includes different types of tasks	98	97	100
Multisensory stimulation	Training that provides more than two types of sensory feedback	83	90	69
Increasing difficulty	Complexity of tasks changes depending on participants’ ability, performance or time	76	76	80
Explicit feedback	Training that provides information about the patient’s performance at the end of the task	79	93	46*
Implicit feedback	Training that delivers information about the performance in real time such as visualization of movement or other kinematic properties	74	83	54
Avatar representation	Embodied training by representation of a human or body part	38	41	31
Use of the paretic limb	Promoting the use of the paretic limb	76	76	80

All studies, 42 included in meta-analysis

+ , studies with SMD in favour of the experimental group for main outcomes regarding upper limb function

= , studies with SMD in favour of the control group for main outcomes regarding upper limb function

*Statistically significant difference (p < 0.05) in Fischer’s exact test

### Outcome measurements

Outcome measures were selected in accordance to the ICF-WHO model [19]. In each category, assessment scales were chosen based on recent literature recommendations [24, 25]. The Fugl-Meyer Assessment (FMA) [26] was used for the body function domain. The Action Research Arm Test (ARAT) [27], the Box and Block Test (BBT) [28] and the Wolf Motor Function Test (WMFT) [29] were used for the activity domain. The social participation subscale of the Stroke Impact Scale (SIS) [30] was used for the participation domain.

When available, mean improvements in terms of change-from-baseline and their standard deviation (SD) were extracted for each time point. If not available, authors were contacted via email. In case of non-response, the mean improvement was calculated through subtraction between post-intervention mean score and pre-intervention mean score. Then, the SD was estimated by using a formula according to the Cochrane Handbook for Systematic Reviews of Interventions [31]. The value of the correlation coefficient was imputed by using data from other studies [17, 32, 33] included in the meta-analysis. Lastly, when only median and quartiles were available, the mean and SD were approximated using the method proposed by Wan et al. [34]. For studies using follow-up evaluations at least one month after the intervention, mean improvements in terms of change-from-baseline were calculated in order to assess long-term effect retention.

### Data and statistical analysis

Articles scoring below 5/10 on the PEDro scale were excluded due to overall poor methodological quality [35]. In addition, only trials that described conventional therapy used in the comparison group as including occupational, physical or self-therapy were considered for statistical analysis. Statistical analyses were performed using the RevMan 5.3 software [36]. Since different rating scales were used for studied outcomes and results were reported in various ways, standardized mean difference (SMD) and 95% confidence interval (CI) were calculated. This method allowed standardization of results across studies. A random effects meta-analysis model was used for all analyses and statistical significance level was set at *P* < 0.05 [37]. Heterogeneity across trials was estimated using the I^2^ test. Heterogeneity was not considered to be significative for a I^2^ < 30% [30].

Subgroup analysis was conducted for RCT whose intervention met at least 8/11 neurorehabilitation principles compared to RCT whose intervention fulfilled less. Another subgroup analysis was performed according to stroke stage, comparing effects of interventions using serious games on subacute and chronic stroke patients. Subgroup analysis were only considered when at least two trials in each subgroup reported a given domain. Furthermore, long-term effect retention for trials that measured outcomes at follow-up was evaluated.

Publication bias was evaluated visually through funnel plot graphic representation. Sensitivity analyses was conducted to verify results robustness in case of funnel plot asymmetry, heterogeneity or presence of outliers. Additional sensitivity analyses were conducted using two different values for correlation coefficient [30]. GRADEpro program was used to assess the strength of the body evidence [38].

Finally, a Fischer’s exact test was used to compare differences in proportions among studies, depending on their results, regarding adherence to each neurorehabilitation principle.

## Results

### Study selection

**Fig. 1:**
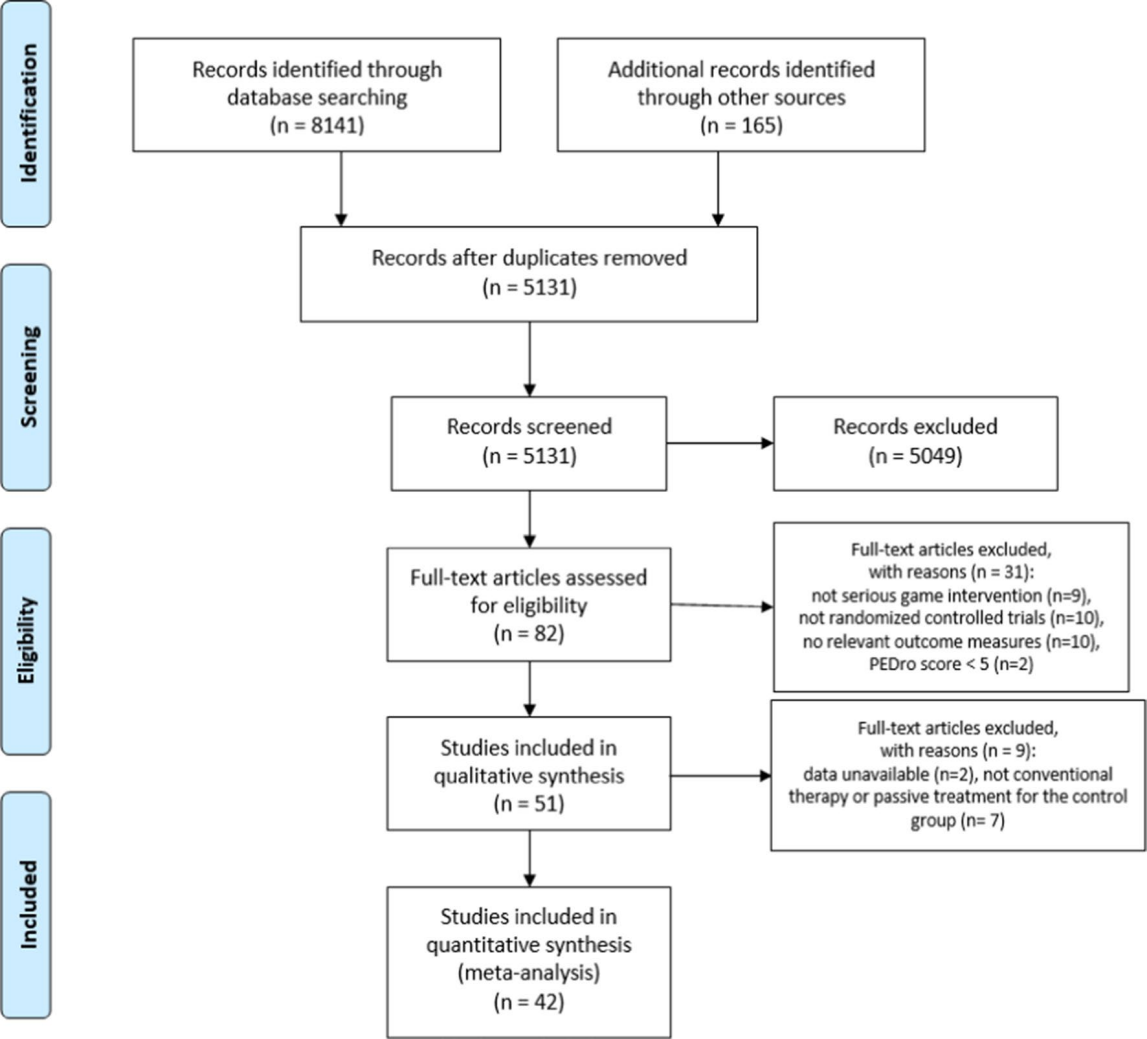
Flow chart (PRISMA) of the selection process

A total of 8141 trials were identified through search across all databases and 165 additional records through other sources. After removal of duplicates, 5131 articles were screened based on titles and abstracts. Among these, 5049 were excluded and 82 full-text articles were assessed for eligibility. 51 RCT were included in the qualitative synthesis. Finally, after quality assessment was performed, 42 RCT were considered for quantitative synthesis. Further details are illustrated in the study PRISMA flow chart (Fig. 1).

### Study characteristics

A total of 2083 participants with a mean age ranging from 49.3 to 76.0 years were included in the qualitative synthesis. For each included study, we identified the mean age of the participants, the stroke stage classification and the type of device used for intervention (Table 2). Approximately one third (31%) of studies included stroke patients at subacute stage and two-thirds (69%) at chronic stage. Across trials, serious games were implemented on different types of devices: 26 (51%) used a motion capture system among which many low-cost systems (such as Microsoft Kinect for example), 10 (19%) used an end-effector type robot, 5 (9%) used motion capture gloves, 3 (7%) a robotic exoskeleton, 3 (6%) an immersive-VR system, 2 (4%) a smartphone or tablet, 1 (2%) a surface EMG-controlled sensor and 1 (2%) an arm support system.

**Table 2:** Characteristics of included studies

Author	Participants^a^	Age^b^	Stroke stage	Type of device	PEDro score
Adomaviciene [51]	42	64.6	Subacute	Motion capture system, LCD monitor	5
Ang [52]	21	54.2	Chronic	Haptic Knob robotic system, LCD monitor	6
Aprile [53]	224	69.5	Subacute	4 different robotic devices	6
Askin [54]	38	55.0	Chronic	Motion capture system, LCD monitor	6
Brunner [40]	120	62.0	Subacute	Motion capture gloves, LCD monitor	7
Cameirao [55]	19	61.0	Subacute	Motion capture system, data gloves, LCD monitor	5
Cameirao [56]	44	62.0	Chronic	Motion capture system, data gloves, LCD monitor	6
Cho [57]	38	60.0	Chronic	End-effector robot, LCD monitor	8
Choi [58]	24	61.0	Subacute	Smartphone and tablet computer	6
Crosbie [59]	18	60.0	Chronic	Immersive VR motion tracking system	8
Dehem [14]	45	67.3	Subacute	End-effector robot, LCD monitor	7
Duff [60]	21	68.5	Chronic	Motion capture system, LCD monitor	5
Henrique [61]	31	76.0	Chronic	Immersive VR motion tracking system	5
Housman [62]	28	55.0	Chronic	Robotic exoskeleton, LCD monitor	5
Hung [13]	33	58.5	Chronic	Motion capture system, LCD monitor	7
Jang [63]	10	57.1	Chronic	Motion capture system, LCD monitor	5
Jo [64]	29	64.0	Chronic	Motion capture system, LCD monitor	5
Kim [65]	23	53.5	Subacute	Motion capture system, LCD monitor	8
Kiper [66]	80	64.0	Subacute	Motion capture system, LCD monitor	5
Kiper [67]	44	64.3	Subacute	Motion capture system, LCD monitor	5
Kiper [46]	136	63.9	Subacute	Motion capture system, LCD monitor	6
Klamroth-Marganska [68]	73	56.5	Chronic	Robotic exoskeleton, LCD monitor	8
Kottink [32]	18	61.5	Chronic	Motion capture system, LCD monitor	6
Kwon [69]	26	57.5	Subacute	Motion capture system, LCD monitor	5
Laffont [44]	51	58.0	Subacute	Touchscreen interface, computer monitor	8
Lee [70]	26	67.5	Chronic	Motion capture system, LCD monitor	8
Lee [71]	18	71.1	Chronic	Motion capture system, LCD monitor	6
Lee [72]	30	51.0	Chronic	End-effector robot, LCD monitor	6
Levin [73]	12	58.5	Chronic	Motion capture system, LCD monitor	6
Liao [74]	20	54.5	Chronic	End-effector robot, LCD monitor	7
Mugler [75]	32	58.0	Chronic	Surface EMG-controlled sensor, computer monitor	6
Nijenhuis [76]	19	60.0	Chronic	Arm support system	6
Norouzi-Gheidari [39]	18	49.9	Chronic	Motion capture system, LCD monitor	7
Ogun [77]	65	60.6	Chronic	Immersive VR motion tracking system	6
Oh [17]	31	55.0	Chronic	3-D manipulator, computer monitor	7
Park [33]	25	52.5	Chronic	2-D planar motion handlebar, LCD monitor	7
Piron [78]	36	65.2	Chronic	Motion capture camera, computer monitor	7
Piron [47]	47	60.5	Chronic	Motion capture system, LCD monitor	8
Prange [79]	68	59.1	Subacute	Arm support system, computer monitor	7
Rogers [80]	21	64.4	Subacute	Touchscreen mega-tablet	6
Schuster-Amft [81]	54	61.3	Chronic	Motion capture gloves, LCD monitor	8
Shin [82]	16	49.3	Subacute	Motion capture system, LCD monitor	5
Shin [83]	32	54.0	Chronic	Motion capture system, LCD monitor	6
Shin [84]	46	58.5	Chronic	Motion capture gloves, LCD monitor	7
Subramanian [85]	32	61.0	Chronic	Motion capture system, LCD monitor	7
Thielbar [86]	14	56.5	Chronic	Pneumatically actuated motion capture gloves	6
Thielbar [87]	20	59.7	Chronic	Motion capture system, LCD monitor	5
Tomic [88]	26	57.4	Subacute	End-effector robot, LCD monitor	7
Wolf [89]	99	56.9	Chronic	End-effector robot, computer touch screen	7
Yin [90]	23	58.3	Subacute	Motion capture system, computer monitor	6
Zondervan [91]	17	59.5	Chronic	Motion capture gloves, computer monitor	6

LCD monitor, liquid–crystal display monitor; 3-D, 3-Dimensional; 2-D, 2-Dimensional

^a^Participants: number of total participants in study

^b^Age: mean age in years estimated for total number of participants included in each study

For each trial, total treatment duration in terms of minutes per session, number of sessions per week and total number of weeks was identified. In addition, whether intervention and control groups were time-matched regarding these characteristics was verified (Table 3). Total number of weeks of treatment varied from 2 to 12 weeks with a mean of 5 weeks among trials. Daily duration of therapy varied widely among studies ranging from 30 to 225 min. In most trials (85%), total treatment duration was matched between the intervention and control groups (Table 3).

**Table 3:** Duration, matched groups, outcome measurements, overall findings, number of included neurorehabilitation principles

Authors and publication year	Duration^a^	Matched groups^b^	UL function	UL activity	Participation	Overall findings^c^	Principles^d^
Adomaviciene, 2019 [51]	2	✓	FMA-UE	BBT		+	4
Ang, 2014 [52]	6	✓	FMA-UE			=	5
Aprile [53]	6	✓	FMA-UE^s^	ARAT^s^		=	10
Askin, 2018 [54]	4	X	FMA-UE^m, s^	BBT^m, s^		+	6
Brunner, 2017 [40]	4	✓		ARAT^s^, BBT		=	4
Cameirao, 2011 [55]	12	✓	FMA-UE			+	6
Cameirao, 2012 [56]	4	✓	FMA-UE	BBT		+	6
Cho, 2019 [57]	6	✓	FMA-UE	ARAT, BBT		+	6
Choi, 2016 [58]	2	✓	FMA-UE ^s^			+	8
Crosbie, 2012 [59]	3	✓		ARAT ^s^		=	6
Dehem, 2019 [14]	9	✓	FMA-UE	BBT	SIS	+	9
Duff, 2013 [60]	4	✓	FMA-UE^m, s^	WMFT^m, s^	SIS	=	9
Henrique, 2019 [61]	12	✓	FMA-UE			+	9
Housman, 2009 [62]	9	✓	FMA-UE			+	5
Hung, 2019 [13]	12	✓	FMA-UE^m, s^	WMFT^m, s^		=	8
Jang, 2005 [63]	4	✓	FMA^s^	BBT^s^		+	10
Jo, 2012 [64]	4	X		WMFT		+	9
Kim, 2018 [65]	2	✓	FMA-UE^s^	BBT^s^		=	7
Kiper, 2011 [66]	4	✓	FMA-UE^s^			+	9
Kiper, 2014 [67]	4	✓	FMA-UE^s^			+	9
Kiper, 2018 [46]	4	✓	FMA-UE^s^			+	8
Klamroth-Marganska, 2014 [68]	8	✓	FMA-UE		SIS	+	7
Kottink, 2014 [32]	6	✓	FMA-UE	ARAT		=	6
Kwon, 2012 [69]	4	X	FMA-UE^s^			=	5
Laffont [44]	6	✓	FMA-UE	BBT, WMFT		=	8
Lee, 2016a [70]	8	✓	FMA-UE^s^	BBT^s^		+	8
Lee, 2016b [71]	6	✓		BBT		+	9
Lee, 2018 [72]	8	✓	FMA-UE^s^			+	8
Levin, 2012 [73]	3	✓	FMA-UE^s^	BBT^s^, WMFT		+	9
Liao, 2012 [74]	4	✓	FMA-UE^s^			+	7
Mugler, 2019 [75]	3	X	FMA-UE			=	8
Nijenhuis, 2017 [76]	6	✓	FMA-UE^m, s^	ARAT^m, s^, BBT	SIS	=	5
Norouzi-Gheidari, 2019 [39]	4	X	FMA-UE^s^	BBT^s^	SIS^s^	+	8
Ogun, 2019 [77]	6	✓	FMA-UE^s^	ARAT^s^		+	8
Oh, 2019 [17]	6	✓	FMA-UE	BBT		+	9
Park, 2019 [33]	4	✓	FMA-UE	WMFT	SIS	=	9
Piron, 2009 [78]	4	✓	FMA-UE^s^			+	8
Piron (2010) [47]	4	✓	FMA-UE^s^			+	10
Prange, 2015, [79]	6	✓		BBT		+	9
Rogers2019 [80]	4	X	FMA-UE^s^			=	5
Schuster-Amft, 2018 [81]	4	✓		BBT^m, s^	SIS	=	7
Shin, 2014 [82]	2	X	FMA-UE^s^			=	8
Shin, 2015 [83]	4	✓	FMA-UE^m, s^			=	9
Shin, 2016 [84]	4	✓	FMA-UE^s^		SIS^s^	+	10
Subramanian, 2012 [85]	4	✓	FMA-UE			+	8
Thielbar, 2014 [86]	6	✓	FMA-UE^s^	ARAT^s^		+	10
Thielbar, 2020 [87]	4	✓	FMA-UE			+	8
Tomic, 2017 [88]	3	✓	FMA-UE	WMFT		+	8
Wolf, 2015 [89]	8	✓	FMA-UE^s^	ARAT^s^, WMFT		=	6
Yin, 2014 [90]	2	✓	FMA-UE^m, s^	ARAT^m, s^		=	11
Zondervan, 2016 [91]	3	✓		ARAT, BBT		=	7

*UL* upper limb, *FMA-UE* Fugl-Meyer Assessment Upper Extremity subscale, *ARAT* action research arm test, *BBT* box and block test, *WMFT* Wolf-motor function test, *SIS* stroke impact scale, ✓, matched time between interventions; X, time between interventions not matched; + , statistically significant improvement in favour of experimental group for main outcomes; = , no statistically significant differences reported between experimental and control group

^a^Duration: total number of treatment weeks

^b^Matched groups: matched time in terms of daily session time, sessions per week and total number of weeks between experimental and control group

^c^Overall findings: reported findings concerning primary outcome measures

^d^Principles: total number of neuro-rehabilitation principles fulfilled by the serious game used in the intervention. A total of 11 principles were examined for each trial

^m^Studies that reported only median and quartiles

^s^Studies for which the standard deviation had to be estimated

The number of neurorehabilitation principles fulfilled by serious games were identified through content analysis. This number varied from 4 to 11 (Table 3). For a total of 11 neurorehabilitation principles, 32 (63%) interventions met 8 or more, 17 (33%) met between 5 and 7 and 2 (4%) interventions met less than 5. Table 1 illustrates the percentage of studies included in meta-analysis that complied with each neurorehabilitation principle. In addition, Table 1 displays differences in adherence to each neurorehabilitation principle between studies with overall positive or negative results (based on each study SMD in quantitative synthesis results). Statistically significant differences were observed regarding the principle of explicit feedback. Indeed, the group of studies with overall positive results adheres more to this principle than the other group.

Regarding main outcomes, 44 trials (87%) assessed UL motor function, 30 (59%) assessed UL activity and 9 (17%) assessed participation (Table 3). Most trials (60%) reported significantly superior results in at least one ICF-WHO component in favour of interventions using serious games compared to conventional treatment.

### Methodological quality and risk of bias assessment

PEDro scores of 51 included studies ranged from 5 to 8 with a mean (SD) of 6.33 (1.15) indicating an overall moderate to high methodological quality (Table 2). Detailed PEDro scale scoring for each trial is illustrated in Additional file 1: Table S1. In addition, the detailed analysis using the Cochrane Collaboration RoB tool is presented in Additional file 1: Fig. S1.

### Effect of rehabilitation through serious games on UL motor function

**Fig. 2:**
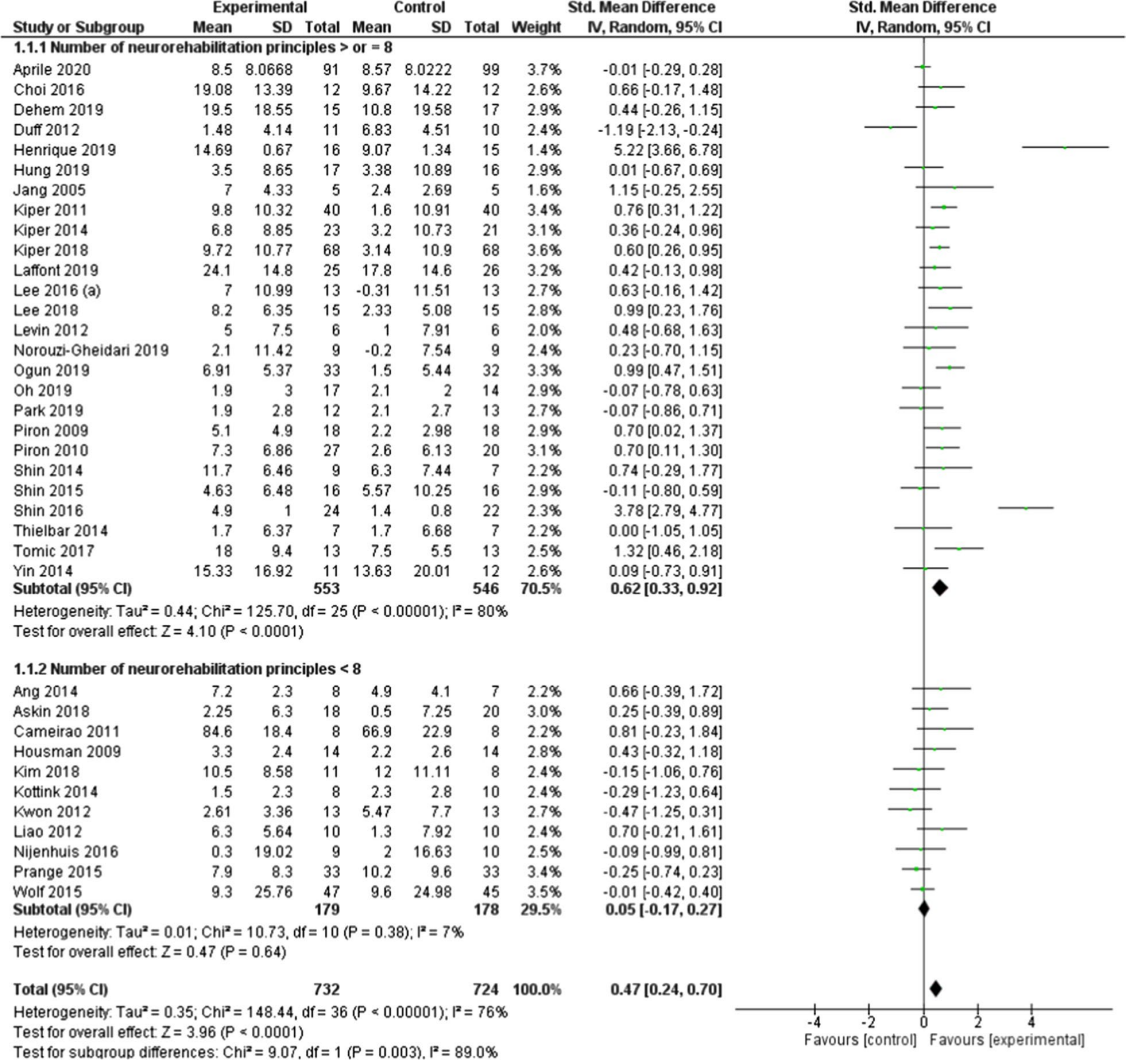
Forest plot of upper limb motor function as measured by the FMA-UE: studies using a serious game fulfilling ≥ 8 Npr versus studies using a serious game fulfilling < 8 Npr. *FMA-UE* upper extremity subscale of the Fugl-Meyer Assessment, *Npr* neurorehabilitation principles

In total, rehabilitation using serious games led to significantly better improvements, of moderate effect size, in UL motor function compared to conventional treatment (SMD = 0.47; 95% CI = 0.24 to 0.70; *P* < 0.0001) (Fig. 2). Subgroup analysis highlighted differences between results of trials using serious games fulfilling 8 or more neurorehabilitation principles and those that did not (*P* = 0.003). Indeed, only interventions that met 8 or more principles showed significant impact of moderate effect size on upper limb motor function (SMD = 0.62; 95% CI = 0.33 to 0.92; *P* = 0.0001). Although total results indicated considerable heterogeneity between studies (I^2^ = 76%), analysis using the GRADE approach led to a moderate certainty of evidence (Additional file 1: Fig. S2 illustrates detailed summary of findings).

**Fig. 3:**
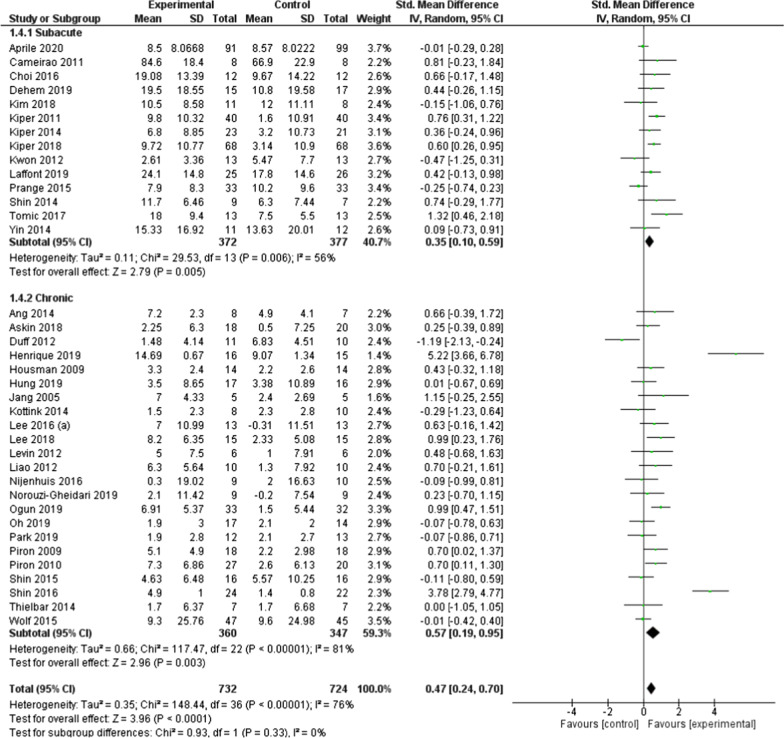
Forest plot of upper limb motor function as measured by the FMA-UE: studies in the subacute phase after stroke versus studies in the chronic phase after stroke. FMA-UE, upper extremity subscale of the Fugl-Meyer Assessment

Additional subgroup analysis was conducted based on the stroke stage of included participants across studies (Fig. 3). Results suggest that interventions using serious games were effective in improving UL motor function in both subacute (SMD = 0.35; 95% CI = 0.10 to 0.59; *P* = 0.006) and chronic stage after stroke (SMD = 0.57; 95% CI = 0.19 to 0.95; *P* = 0.003). Differences among subgroups did not reach statistical significance (*P* = 0.33).

Finally, in order to address heterogeneity, sensitivity analyses were performed in two ways. A first analysis was conducted by excluding outliers identified through funnel plot graphic representation (Additional file 1: Fig. S3). Then, a second analysis was carried out by using a different correlation coefficient value. In both cases, results indicate no significant differences in total estimates when compared to initial findings (Additional file 1: Figs. S4 and S5).

### Effect of rehabilitation through serious games on UL activity

**Fig. 4:**
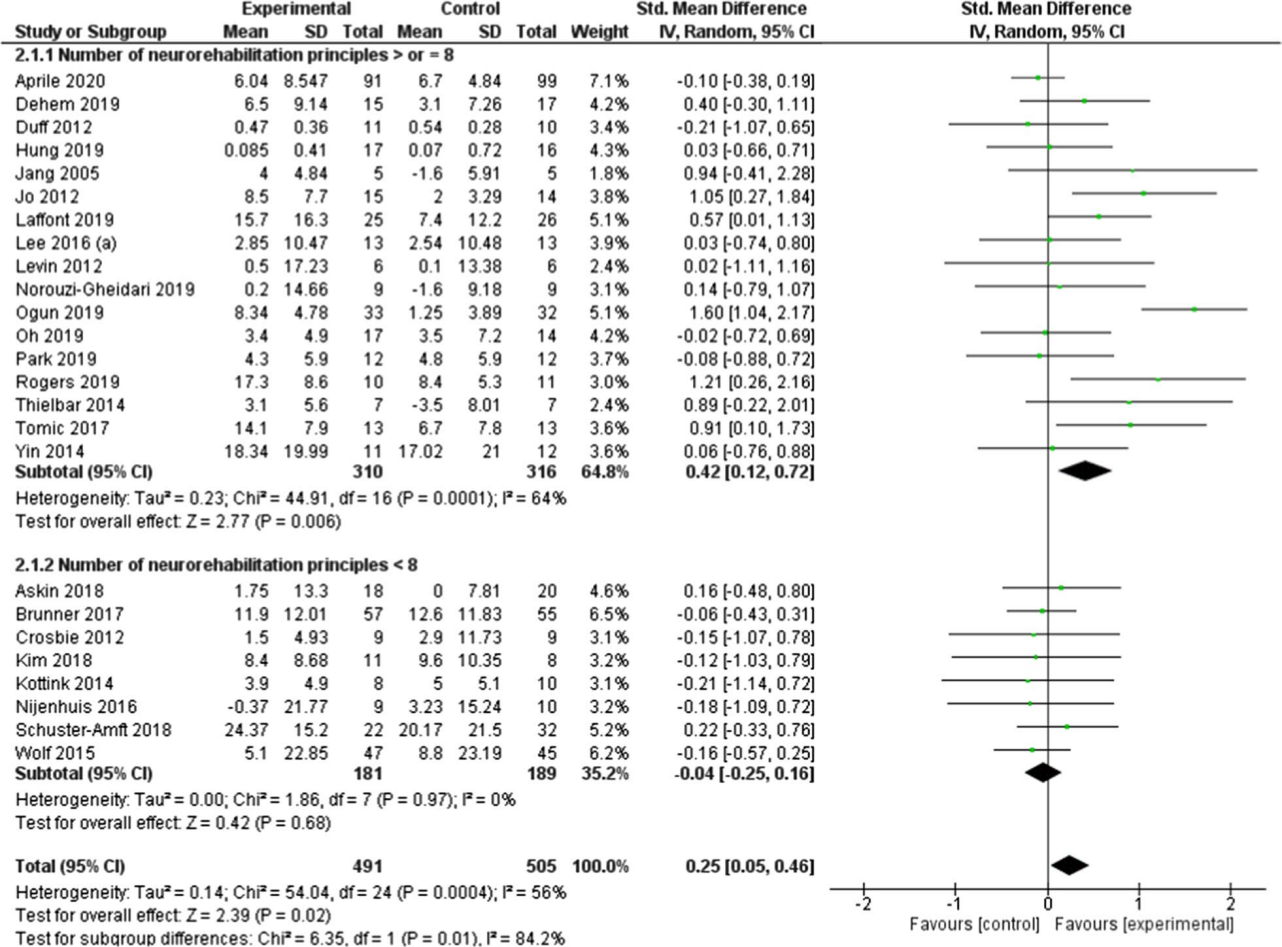
Forest plot of upper limb activity as measured by the ARAT, BBT, WMFT: studies using a serious game fulfilling ≥ 8 Npr versus studies using a serious game fulfilling < 8 Npr. *ARAT* action research arm test, *BBT* box and block test, *WMFT* Wolf motor function test, *Npr* neurorehabilitation principles

In total, rehabilitation using serious games led to significantly better improvements, of low effect size, in upper limb activity compared to conventional treatment (SMD = 0.25; 95% CI = 0.05 to 0.46; *P* = 0.02) (Fig. 4). In a similar way to results regarding UL function, subgroup analysis showed significantly better improvements, of moderate effect size, only for interventions that fulfilled 8 or more neurorehabilitation principles (SMD = 0.42; 95% CI = 0.12 to 0.72; *P* = 0.006). Differences among subgroups were statistically significant (*P* = 0.01). Total results indicated moderate heterogeneity between studies (I^2^ = 56%). Additional subgroup analysis based on stroke stage did not reach statistical significance for neither subacute or chronic stage after stroke (Additional file 1: Fig. S6).

### Effect of rehabilitation through serious games on participation

**Fig. 5:**
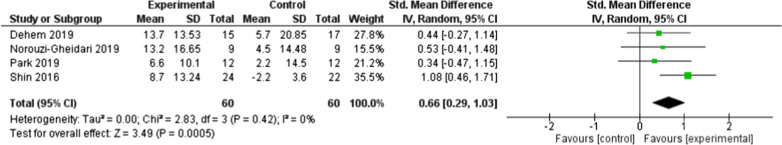
Forest plot of participation as measured by the social participation subscale of the SIS. *SIS* stroke impact scale

In total, rehabilitation using serious games led to significantly better improvements, of large effect size, in participation compared to conventional treatment (SMD = 0.66; 95% CI = 0.29 to 1.03; *P* = 0.0005) (Fig. 5). No significant heterogeneity was present (I^2^ = 0%). All trials included in this analysis used a serious game that complied with 8 or more neurorehabilitation principles.

### Analysis of follow-up data

Separate analyses were conducted regarding follow-up data for each ICF-WHO component. Only half of the studies included in the quantitative synthesis (50%) performed follow-up evaluations. Among them, length of follow-up period ranged from 1 to 6 months with a mean (SD) of 2.3 months (1.86). An overall tendency towards improvement for interventions using serious games regarding all ICF-WHO components was observed (Additional file 1: Figs. S7, S8 and S9). Total estimates concerning UL function indicate effect retention to follow-up in favour of the experimental group of moderate effect size (SMD = 0.42; 95% CI = 0.05 to 0.79; *P* = 0.03). Results did not reach statistical significance regarding UL activity and participation.

## Discussion

### Main results

This systematic review and meta-analysis showed results in favour of rehabilitation using, purpose-built, serious games on UL motor function, UL activity and participation after stroke compared to conventional treatment. Moreover, long term effect retention was significantly maintained regarding UL function. Irrespective of the technological device used, serious games that complied with more than 8 out of 11 neurorehabilitation principles showed better overall effects.

### Previous studies on effectiveness of VRS/CVG for UL rehabilitation after stroke

Previous work on the use of VRS and CVG for UL rehabilitation after stroke demonstrated similar results [11, 17]. Yet, to date, usage and efficacy of game-based interventions for UL rehabilitation after stroke remain controversial [38–40]. Initially, a meta-analysis by Saposnik et al., combining observational studies and RCT, suggested improvements in UL strength and motor function after stroke [41]. However, this review focused on various VRS, including CVG designed by the entertainment industry, not specifically developed for rehabilitation. In addition, no statistically significant differences were observed concerning UL activity outcomes and no analysis was conducted regarding ICF-WHO participation component due to limited available data.

Two other groups conducted systematic reviews on a similar topic [42, 43]. However, both reviews included studies concerning not only UL rehabilitation but also gait and balance, making it difficult to draw conclusions regarding the UL. Palma et al., solely relying on qualitative synthesis, supported positive findings on function [42]. Results were inconclusive regarding activity and participation components and further interpretation was limited due to lack of quantitative synthesis. Then, a meta-analysis by Lohse et al. showed positive effects in favour of VR-based interventions regarding three ICF-WHO components [43]. However, analysis was restricted to therapies that did not include robotic assistance. Furthermore, analyses through meta-regressions did not point out significant differences in outcomes between commercially available and custom-built systems.

Two updated Cochrane reviews covered broader aspects of VR and robotics in UL rehabilitation after stroke [8, 10]. In the review by Mehrholz et al., high quality evidence supports better improvements in ADL, arm function and arm strength in favour of RAT [8]. Nonetheless, effects of robotic training performed in form of a serious game were not studied. Then, a review by Laver et al. on VR-based interventions, demonstrated equivalent improvements in UL function and activity when comparing time-matched interventions [10]. Notably, UL function and activity outcomes were pooled in one common analysis instead of distinguishing effects in terms of the two ICF-WHO components. Further analyses in subgroups suggested better results when specific systems designed for rehabilitation were employed compared to off-the-shelf CVG, although differences did not reach statistical significance.

Finally, two recent reviews showed improvements on both UL function and activity in groups receiving VR/gaming-based training after stroke [11, 18]. However, both reviews studied broader aspects of VR-based interventions and their scope was not delimited to specific use of serious games. Karamians et al. suggested that interventions with gaming components further promote recovery compared to those providing visual feedback only [18]. Then, Maier et al. distinguished VRS specifically built for rehabilitation purposes from others destined to generic use [11]. Results illustrated that, when compared to conventional therapy, interventions specifically designed based on elements enhancing neural plasticity led to significantly better results [11]. Additionally, it was suggested that custom-made interventions, in comparison to non-specific interventions, comply better with a series of neurorehabilitation principles.

### Adherence to neurorehabilitation principles of interventions using serious games for UL rehabilitation after stroke

To this date, UL stroke recovery through games developed specifically for rehabilitation and implemented on diverse systems, has not been explicitly reviewed. In addition, most recent reviews delimit their scope in technological terms by considering interventions based on the devices being used [11, 12, 18]. Some authors characterise comparison between studies using different devices as difficult [44]. However, a holistic overview of serious games, regardless of the technology used, is important in order to better understand their added value in UL rehabilitation after stroke. Comparison between studies using systems with different technical specificities, mainly in hardware, is challenging. Nonetheless, interventions through serious games implemented on different devices may share similarities. Indeed, all studies included in our review perform non-invasive treatments. Then, gamification and adaptability of interventions, to the patients’ impairments and performance, aim to maintain motivation throughout therapy sessions [45]. Additionally, all these systems have the potential to give access to kinematic data allowing objective assessment, evaluating real-time performance and tracking UL recovery [46–48]. Finally, all interventions stimulate recovery through adherence to common neurorehabilitation principles. In fact, comparison across different types of technologies and treatment modalities leads to identification of common ‘active ingredients’ in terms of effective rehabilitation [11]. In accordance with recent literature, this review contributes to identifying a rationale regarding efficacy of interventions in UL rehabilitation after stroke. Our results point out that even in a group of interventions specifically developed for rehabilitation purposes, differences in outcomes may be explained depending on higher adherence to neurorehabilitation principles. Furthermore, even though most interventions seem to fulfil certain principles (task-specific practice, variable practice, massed practice), it seems that clusters of principles met among serious games may lead to differences in efficacy. For instance, our findings suggest that providing feedback during therapy appears to be an important characteristic that interventions using serious games should satisfy. Further, to what degree each individual principle contributes in efficacy is difficult to study. However, it appears that the more an intervention adheres to principles, the better the expected outcomes can be regarding motor recovery.

To the best of our knowledge, this systematic review is the first to address, in a non-fragmented way, efficacy of specifically designed gaming interventions in UL rehabilitation after stroke. Our results confirm current trends favouring custom-made rehabilitation systems and gamification of interventions. Positive findings concerning function and activity have already been reported in previous reviews [11, 18]. It is worth noting that this review shows encouraging results in participation outcomes indicating, therefore, improvements in three ICF-WHO components.

### Strengths and limitations

In a rapidly emerging field, 40% of studies included in our review were published within the last 3 years. Quantitative synthesis was performed by only using RCT of moderate to high methodological quality. However, this was not feasible for two studies due to unavailable data. Additionally, even though our work was conducted according to PRISMA guidelines for systematic reviews, no methods were used to detect unpublished trials. Also, publication bias was only assessed through funnel plot graphic representation which nonetheless did not indicate asymmetry. Heterogeneity across studies was moderate to high regarding UL function and activity outcomes. This may be partially due to variation of elements such as patient characteristics, duration of interventions and evaluation timepoints. Heterogeneity was addressed by using a random effects model for meta-analyses and by conducting additional analyses. Even though heterogeneity levels remained moderate, our results were little affected by changes in methods or outliers, indicating robustness.

### Perspectives

Our work offers some suggestions regarding clinical practice and future research. Interventions using serious games may be encouraged and integrated in upper limb rehabilitation programs during subacute and chronic stage after stroke. Specifications regarding dosage, duration and selection of patients that could benefit most from these treatments need further investigation. In addition, serious games should be explored in terms of ways to provide self- or tele-rehabilitation.

From a research point of view, new developments in gaming interventions can take into consideration adherence to neurorehabilitation principles. In accordance with our findings, future developments of interventions in UL stroke rehabilitation ought to comply with as many neurorehabilitation principles as possible. Future work should study how variations in clusters of these principles may influence differently specific aspects of motor or cognitive rehabilitation. Also, richness of kinematic data, accessible through technological devices on which games are implemented, open new perspectives in assessment and follow-up of stroke patients. In our review, only 11% of studies used kinematic data, complementary to clinical rating scales, for UL function evaluation. Finally, few studies (11%) included in our review reported cognitive outcomes. Since motor performance and functional recovery can be influenced by cognitive determinants [49, 50], combined assessment of all these aspects should be further considered in future work.

## Conclusion

In conclusion, this systematic review and meta-analysis showed that post-stroke UL rehabilitation through serious games, implemented on various types of technological devices, showed better improvements, compared to conventional treatment, on three ICF-WHO components. Long term effect retention was maintained for UL function. Irrespective of the technological system used, serious games that complied with more than 8 out of 11 neurorehabilitation principles led to better overall effects. Our findings emphasize the importance of adherence to neurorehabilitation principles in order to improve efficacy of interventions in UL rehabilitation after stroke.

## Supplementary Information

**Additional file 1: Table S1.** Detailed PEDro scale scoring for each study. **Figure S1.** Detailed analysis using the Cochrane collaboration risk of bias tool. **Figure S2.** Detailed summary of findings using the GRADEpro approach. **Figure S3.** Funnel plot graphical representation. **Figure S4.** Sensitivity analysis without outliers. **Figure S5.** Sensitivity analysis: use of different correlation coefficient value (0.9). Forest plot of upper limb motor function as measured by the FMA-UE: studies using a serious game fulfilling ≥ 8 Npr versus studies using a serious game fulfilling < 8 Npr. Abbreviations; FMA-UE, upper extremity subscale of the Fugl Meyer Assessment; Npr, Neurorehabilitation principles. **Figure S6.** Forest plot of upper limb activity as measured by the ARAT, BBT, WMFT: studies in the subacute phase after stroke versus studies in the chronic phase after stroke. Abbreviations; ARAT, Action Research Arm Test; BBT, Box and Block test; WMFT, Wolf Motor Function Test; Npr, Neurorehabilitation principles. **Figure S7.** Follow-up evaluation. Forest plot of upper limb motor function as measured by the FMA-UE: studies using a serious game fulfilling ≥ 8 Npr versus studies using a serious game fulfilling < 8 Npr. Abbreviations; FMA-UE, upper extremity subscale of the Fugl Meyer Assessment; Npr, Neurorehabilitation principles. **Figure S8.** Follow-up evaluation. Forest plot of upper limb activity as measured by the ARAT, BBT, WMFT: studies using a serious game fulfilling ≥ 8 Npr versus studies using a serious game fulfilling < 8 Npr. Abbreviations; ARAT, Action Research Arm Test; BBT, Box and Block test; WMFT, Wolf Motor Function Test; Npr, Neurorehabilitation principles. **Figure S9.** Follow-up evaluation. Forest plot of participation as measured by the social participation subscale of the SIS. Abbreviations; SIS, Stroke Impact Scale.

## Data Availability

All data generated or analysed during this study are included in this published article and its supplementary information files.

## Abbreviations

UL: Upper limb
ADL: Activities of daily living
VR: Virtual reality
VRS: Virtual reality systems
RAT: Robot-assisted therapy
CVG: Commercial video-games
ICF-WHO: World Health Organization’s International Classification of Function, Disability, and Health
RCT: Randomized controlled trials
FMA: Fugl-Meyer assessment
ARAT: Action Research Arm Test
BBT: Box and block test
SIS: Stroke impact scale
SMD: Standardized mean difference
CI: Confidence interval
