# Supplementary material for: Serious games for upper limb rehabilitation after stroke: a meta-analysis
Source: J Neuroeng Rehabil. 2021 Jun 15;18:100. doi: 10.1186/s12984-021-00889-1 (PMC8204490; doi:10.1186/s12984-021-00889-1)

# ADDITIONAL FILE 1:

## Search strategies:

| | |
|---|---|
| PUBMED | (((((stroke[MeSH Terms]) OR (stroke*[Title/Abstract] OR CVA[Title/Abstract] OR cerebrovascular accident*[Title/Abstract] OR brain vascular accident*[Title/Abstract] OR brain ischaemic attack*[Title/Abstract] OR brain ischemic attack*[Title/Abstract] OR brain vascular accident*[Title/Abstract] OR cerebral vascular accident*[Title/Abstract] OR cerebro vascular accident*[Title/Abstract] OR cerebrovascular accident*[Title/Abstract] OR "cerebrovascular arrest"[Title/Abstract] OR "cerebrovascular failure"[Title/Abstract] OR cerebrum vascular accident*[Title/Abstract] OR ischaemic cerebral attack*[Title/Abstract] OR ischemic cerebral attack*[Title/Abstract]))) AND ((adult[MeSH Terms]) OR adult*[Title/Abstract])) AND ((((((((video games[MeSH Terms]) OR mobile applications[MeSH Terms]) OR virtual reality[MeSH Terms]) OR virtual reality exposure therapy[MeSH Terms]) OR therapy, computer assisted[MeSH Terms]) OR artificial intelligence[MeSH Terms]) OR robotics[MeSH Terms]) OR (serious game*[Title/Abstract] OR video game*[Title/Abstract] OR computer game*[Title/Abstract] OR TV game*[Title/Abstract] OR television game*[Title/Abstract] OR "portable software app"[Title/Abstract] OR "portable software apps"[Title/Abstract] OR portable software application*[Title/Abstract] OR mobile application*[Title/Abstract] OR "mobile app"[Title/Abstract] OR "mobile apps"[Title/Abstract] OR "app"[Title/Abstract] OR "apps"[Title/Abstract] OR "Amadeo" [Title/Abstract] OR "AutoAmbulator"[Title/Abstract] OR "Bi-Manu-Track"[Title/Abstract] OR "InMotion"[Title/Abstract] OR "MIME"[Title/Abstract] OR "MIT-Manus"[Title/Abstract] OR "Ne-Re-Bot"[Title/Abstract] OR virtual realit*[Title/Abstract] OR immersive[Title/Abstract] OR interactive[Title/Abstract] OR computer assisted therap*[Title/Abstract] OR robot assisted therap*[Title/Abstract] OR robot assisted rehabilitation*[Title/Abstract] OR exergam*[Title/Abstract]))) |
| EMBASE | ('cerebrovascular accident'/exp OR 'cva' OR 'accident, cerebrovascular' OR 'acute cerebrovascular lesion' OR 'acute focal cerebral vasculopathy' OR 'acute stroke' OR 'apoplectic stroke' OR 'apoplexia' OR 'apoplexy' OR 'blood flow disturbance, brain' OR 'brain accident' OR 'brain attack' OR 'brain blood flow disturbance' OR 'brain insult' OR 'brain insultus' OR 'brain ischaemic attack' OR 'brain ischemic attack' OR 'brain vascular accident' OR 'cerebral apoplexia' OR 'cerebral insult' OR 'cerebral stroke' OR 'cerebral vascular accident' OR 'cerebral vascular insufficiency' OR 'cerebro vascular accident' OR 'cerebrovascular accident' OR 'cerebrovascular arrest' OR 'cerebrovascular failure' OR 'cerebrovascular injury' OR 'cerebrovascular insufficiency' OR 'cerebrovascular insult' OR 'cerebrum vascular accident' OR 'cryptogenic stroke' OR 'ischaemic cerebral attack' OR 'ischaemic seizure' OR 'ischemic cerebral attack' OR 'ischemic seizure' OR 'stroke') AND ('virtual reality'/exp OR 'reality, virtual' OR 'virtual reality' OR 'virtual reality exposure therapy'/exp OR 'virtual reality exposure therapy' OR 'video game'/exp OR 'tv games' OR 'computer game' OR 'computergame' OR 'television game' OR 'video game' OR 'video games' OR 'videogame' OR 'videogames' OR 'mobile application'/exp OR 'mobile app' OR 'mobile application' OR 'mobile applications' OR 'mobile apps' OR 'portable software app' OR 'portable software application' OR 'portable software applications' OR 'portable software apps' OR 'computer assisted therapy'/exp OR 'computer assisted therapy' OR 'therapy, computer-assisted' OR 'rehabilitation robot'/exp OR 'amadeo (device)' OR 'autoambulator' OR 'bi-manu-track' OR 'inmotion' OR 'inmotion arm' OR 'inmotion hand' OR 'inmotion shoulder-elbow' OR 'inmotion wrist' OR 'inmotion2' OR 'inmotion2 arm' OR 'inmotion2 shoulder-elbow' OR 'mime (device)' OR 'mit-manus' OR 'nerebot' OR 'rehabilitation robot' OR 'robot-assisted rehabilitation device' OR 'robotic rehabilitation device' OR 'serious game'/exp OR 'exergaming'/exp OR 'exergame'/exp) |

| | |
|---|---|
| SCOPUS | (TITLE-ABS-KEY (stroke) OR TITLE-ABS (cva) OR TITLE-ABS ( "cerebrovascular accident*" ) OR TITLE-ABS ( "brain vascular accident*" ) OR TITLE-ABS ( "cerebral vascular accident*" ) OR TITLE-ABS ( "cerebro vascular accident*" ) OR TITLE-ABS ( {cerebrovascular arrest} ) OR TITLE-ABS ( {cerebrovascular failure} ) OR TITLE-ABS ( "cerebrum vascular accident*" ) OR TITLE-ABS ( "isch*emic cerebral attack*" ) OR TITLE-ABS ( "brain isch*mic attack*" ) ) AND (TITLE-ABS-KEY ( "video gam*" ) OR TITLE-ABS-KEY ( {virtual reality} ) OR TITLE-ABS-KEY ( {therapy computer assisted} ) OR TITLE-ABS-KEY ( {artificial intelligence} ) OR TITLE-ABS-KEY (robotics ) OR TITLE-ABS-KEY ( {serious game} ) OR TITLE-ABS-KEY ( {serious games} ) OR TITLE-ABS ( "computer game*" ) OR TITLE-ABS ( "mobile application*" ) OR TITLE-ABS (app ) OR TITLE-ABS (apps ) OR TITLE-ABS ( "virtual realit*" ) OR TITLE-ABS (immersive ) OR TITLE-ABS (interactive ) OR TITLE-ABS ( "computer assisted therap*" ) OR TITLE-ABS ( "robot assisted therap*" ) OR TITLE-ABS ( "robot assisted rehabilitation*" ) OR TITLE-ABS ( "exergam*" ) OR TITLE-ABS ( "television gam*" ) OR TITLE-ABS ( "TV gam*" ) OR TITLE-ABS ( "portable software application*" ) OR TITLE-ABS ( {Amadeo} ) OR TITLE-ABS ( {AutoAmbulator} ) OR TITLE-ABS ( {Bi-Manu-Track} ) OR TITLE-ABS ( {InMotion} ) OR TITLE-ABS ( {MIME} ) OR TITLE-ABS ( {MIT-Manus} ) OR TITLE-ABS ( {Ne-Re-Bot} ) ) AND (TITLE-ABS-KEY (adult) ) |
| COCHRANE LIBRARY | ***Title – Abstract - Keywords***<br>stroke OR CVA OR "cerebrovascular accident" OR "brain vascular accident" OR "brain ischaemic attack" OR "brain ischemic attack" OR "brain vascular accident" OR "cerebral vascular accident" OR "cerebro vascular accident" OR "cerebrovascular failure" OR "cerebrovascular arrest" OR "cerebrum vascular accident" OR "ischaemic cerebral attack"<br><br>**AND**<br>***Title – Abstract - Keywords***<br>adult OR adults<br><br>**AND**<br>***Title – Abstract - Keywords***<br>"Virtual reality" OR "Virtual reality exposure therapy" OR "Video game" OR "therapy computer assisted" OR "artificial intelligence" OR robotics OR "Mobile application" OR "Computer assisted therapy" OR "computer game" OR app OR "mobile app" OR "mobile applications" OR "Rehabilitation robot" OR "Serious game" OR "Robot assisted therapy" OR "exergame" OR "exergaming" OR immersive OR interactive OR "computer assisted therapy" OR "television game" OR "TV game" OR "portable software app" OR "portable software application" OR "Amadeo" OR "AutoAmbulator" OR "Bi-Manu-Track" OR "InMotion" OR "MIME" OR "MIT-Manus" OR "Ne-Re-Bot" |

# PRISMA 2009 Checklist

| Section/topic | # | Checklist item | Reported on page # |
|---|---|---|---|
| **TITLE** | | | |
| Title | 1 | Identify the report as a systematic review, meta-analysis, or both. | 1 |
| **ABSTRACT** | | | |
| Structured summary | 2 | Provide a structured summary including, as applicable: background; objectives; data sources; study eligibility criteria, participants, and interventions; study appraisal and synthesis methods; results; limitations; conclusions and implications of key findings; systematic review registration number. | 2-3 |
| **INTRODUCTION** | | | |
| Rationale | 3 | Describe the rationale for the review in the context of what is already known. | 3-4 |
| Objectives | 4 | Provide an explicit statement of questions being addressed with reference to participants, interventions, comparisons, outcomes, and study design (PICOS). | 5 |
| **METHODS** | | | |
| Protocol and registration | 5 | Indicate if a review protocol exists, if and where it can be accessed (e.g., Web address), and, if available, provide registration information including registration number. | 5 |
| Eligibility criteria | 6 | Specify study characteristics (e.g., PICOS, length of follow-up) and report characteristics (e.g., years considered, language, publication status) used as criteria for eligibility, giving rationale. | 6 |
| Information sources | 7 | Describe all information sources (e.g., databases with dates of coverage, contact with study authors to identify additional studies) in the search and date last searched. | 5-6 |
| Search | 8 | Present full electronic search strategy for at least one database, including any limits used, such that it could be repeated. | Supplementary material |
| Study selection | 9 | State the process for selecting studies (i.e., screening, eligibility, included in systematic review, and, if applicable, included in the meta-analysis). | 5-6 |
| Data collection process | 10 | Describe method of data extraction from reports (e.g., piloted forms, independently, in duplicate) and any processes for obtaining and confirming data from investigators. | 6-7 |
| Data items | 11 | List and define all variables for which data were sought (e.g., PICOS, funding sources) and any assumptions and simplifications made. | 6-8 |
| Risk of bias in individual studies | 12 | Describe methods used for assessing risk of bias of individual studies (including specification of whether this was done at the study or outcome level), and how this information is to be used in any data synthesis. | 6-8 |
| Summary measures | 13 | State the principal summary measures (e.g., risk ratio, difference in means). | 8 |
| Synthesis of results | 14 | Describe the methods of handling data and combining results of studies, if done, including measures of consistency (e.g., $I^2$) for each meta-analysis. | 8 |

# PRISMA 2009 Checklist

| Section/topic | # | Checklist item | Reported on page # |
|---|---|---|---|
| Risk of bias across studies | 15 | Specify any assessment of risk of bias that may affect the cumulative evidence (e.g., publication bias, selective reporting within studies). | 8 |
| Additional analyses | 16 | Describe methods of additional analyses (e.g., sensitivity or subgroup analyses, meta-regression), if done, indicating which were pre-specified. | 8-9 |
| **RESULTS** | | | |
| Study selection | 17 | Give numbers of studies screened, assessed for eligibility, and included in the review, with reasons for exclusions at each stage, ideally with a flow diagram. | 9<br>Figure 1 |
| Study characteristics | 18 | For each study, present characteristics for which data were extracted (e.g., study size, PICOS, follow-up period) and provide the citations. | 9-10<br>Table 2 & 3 |
| Risk of bias within studies | 19 | Present data on risk of bias of each study and, if available, any outcome level assessment (see item 12). | 11<br>Supplementary Figure 1 |
| Results of individual studies | 20 | For all outcomes considered (benefits or harms), present, for each study: (a) simple summary data for each intervention group (b) effect estimates and confidence intervals, ideally with a forest plot. | 11-13<br>Figures 2, 3, 4, 5 |
| Synthesis of results | 21 | Present results of each meta-analysis done, including confidence intervals and measures of consistency. | 11-13<br>Figures 2, 3, 4, 5 |
| Risk of bias across studies | 22 | Present results of any assessment of risk of bias across studies (see Item 15). | 11-13<br>Supplementary Figure 3 |
| Additional analysis | 23 | Give results of additional analyses, if done (e.g., sensitivity or subgroup analyses, meta-regression [see Item 16]). | 11-13<br>Supplementary Figures 4, 5, 6, 7, 8, 9 |
| **DISCUSSION** | | | |
| Summary of evidence | 24 | Summarize the main findings including the strength of evidence for each main outcome; consider their relevance to key groups (e.g., healthcare providers, users, and policy makers). | 13 |

| | | | |
|---|---|---|---|
| Limitations | 25 | Discuss limitations at study and outcome level (e.g., risk of bias), and at review-level (e.g., incomplete retrieval of identified research, reporting bias). | 16-17 |
| Conclusions | 26 | Provide a general interpretation of the results in the context of other evidence, and implications for future research. | 17-18 |
| **FUNDING** | | | |
| Funding | 27 | Describe sources of funding for the systematic review and other support (e.g., supply of data); role of funders for the systematic review. | 20 |

*From:* Moher D, Liberati A, Tetzlaff J, Altman DG, The PRISMA Group (2009). Preferred Reporting Items for Systematic Reviews and Meta-Analyses: The PRISMA Statement. PLoS Med 6(7): e1000097. doi:10.1371/journal.pmed1000097

For more information, visit: **www.prisma-statement.org**.

**Table S1.** Detailed PEDro scale scoring for each study

| STUDY | TOTAL | Item 1 | Item 2 | Item 3 | Item 4 | Item 5 | Item 6 | Item 7 | Item 8 | Item 9 | Item 10 | Item 11 |
|---|---|---|---|---|---|---|---|---|---|---|---|---|
| Adomaviciene 2019 | 5 | 1 | 1 | 0 | 1 | 0 | 0 | 0 | 1 | 0 | 1 | 1 |
| Ang 2014 | 6 | 1 | 1 | 0 | 1 | 0 | 0 | 1 | 1 | 0 | 1 | 1 |
| Aprile 2020 | 6 | 1 | 1 | 0 | 1 | 0 | 0 | 1 | 0 | 1 | 1 | 1 |
| Askin 2018 | 6 | 0 | 1 | 0 | 1 | 0 | 0 | 1 | 1 | 0 | 1 | 1 |
| Brunner 2017 | 7 | 1 | 1 | 1 | 1 | 0 | 0 | 1 | 1 | 0 | 1 | 1 |
| Cameirao 2011 | 5 | 1 | 1 | 0 | 1 | 0 | 0 | 1 | 0 | 0 | 1 | 1 |
| Cameirao 2012 | 6 | 1 | 1 | 0 | 1 | 0 | 0 | 1 | 1 | 0 | 1 | 1 |
| Cho 2019 | 8 | 0 | 1 | 1 | 1 | 0 | 0 | 1 | 1 | 1 | 1 | 1 |
| Choi 2016 | 6 | 1 | 1 | 0 | 1 | 0 | 0 | 1 | 1 | 0 | 1 | 1 |
| Crosbie 2012 | 8 | 1 | 1 | 1 | 1 | 0 | 0 | 1 | 1 | 1 | 1 | 1 |
| Dehem 2019 | 7 | 1 | 1 | 1 | 1 | 0 | 0 | 1 | 0 | 1 | 1 | 1 |
| Duff 2012 | 5 | 1 | 1 | 0 | 1 | 0 | 0 | 1 | 0 | 0 | 1 | 1 |
| Faria 2018 | 4 | 1 | 1 | 0 | 0 | 0 | 0 | 0 | 1 | 0 | 1 | 1 |
| Henrique 2019 | 5 | 0 | 1 | 0 | 1 | 0 | 0 | 0 | 1 | 0 | 1 | 1 |
| Housman 2009 | 5 | 1 | 1 | 0 | 1 | 0 | 0 | 1 | 0 | 0 | 1 | 1 |
| Hung 2019 | 7 | 1 | 1 | 0 | 1 | 0 | 0 | 1 | 1 | 1 | 1 | 1 |
| Jang 2005 | 5 | 0 | 1 | 0 | 1 | 0 | 0 | 0 | 1 | 0 | 1 | 1 |
| Jo 2012 | 5 | 1 | 1 | 0 | 1 | 0 | 0 | 0 | 1 | 0 | 1 | 1 |
| Kim 2013 | 3 | 0 | 1 | 0 | 0 | 0 | 0 | 0 | 0 | 0 | 1 | 1 |
| Kim 2018 | 8 | 0 | 1 | 1 | 1 | 1 | 0 | 1 | 0 | 1 | 1 | 1 |
| Kiper 2011 | 5 | 1 | 1 | 0 | 1 | 0 | 0 | 0 | 1 | 0 | 1 | 1 |
| Kiper 2014 | 5 | 1 | 1 | 0 | 1 | 0 | 0 | 0 | 1 | 0 | 1 | 1 |
| Kiper 2018 | 6 | 1 | 1 | 1 | 1 | 0 | 0 | 0 | 1 | 0 | 1 | 1 |
| Klamroth-Marganska 2014 | 8 | 1 | 1 | 1 | 1 | 0 | 0 | 1 | 1 | 1 | 1 | 1 |
| Kottink 2014 | 6 | 1 | 1 | 0 | 1 | 0 | 0 | 1 | 1 | 0 | 1 | 1 |
| Kwon 2012 | 5 | 1 | 1 | 0 | 1 | 0 | 0 | 1 | 0 | 0 | 1 | 1 |
| Laffont 2019 | 8 | 1 | 1 | 1 | 1 | 0 | 0 | 1 | 1 | 1 | 1 | 1 |
| Lee 2016 (a) | 8 | 1 | 1 | 1 | 1 | 0 | 0 | 1 | 1 | 1 | 1 | 1 |
| Lee 2016 (b) | 6 | 1 | 1 | 0 | 1 | 0 | 0 | 1 | 1 | 0 | 1 | 1 |

**Table S1.** (continued)

| | | | | | | | | | | | | |
|---|---|---|---|---|---|---|---|---|---|---|---|---|
| Lee 2018 | 6 | 1 | 1 | 0 | 1 | 0 | 0 | 1 | 1 | 0 | 1 | 1 |
| Levin 2012 | 6 | 1 | 1 | 0 | 1 | 0 | 0 | 1 | 1 | 0 | 1 | 1 |
| Liao 2012 | 7 | 1 | 1 | 1 | 1 | 0 | 0 | 1 | 1 | 0 | 1 | 1 |
| Mugler 2019 | 6 | 0 | 1 | 0 | 1 | 0 | 0 | 1 | 1 | 0 | 1 | 1 |
| Nijenhuis 2016 | 6 | 1 | 1 | 1 | 1 | 0 | 0 | 0 | 1 | 0 | 1 | 1 |
| Norouzi-Gheidari 2019 | 7 | 1 | 1 | 0 | 1 | 0 | 0 | 1 | 1 | 0 | 1 | 1 |
| Ogun 2019 | 6 | 1 | 1 | 0 | 1 | 1 | 0 | 1 | 0 | 0 | 1 | 1 |
| Oh 2019 | 7 | 1 | 1 | 1 | 1 | 0 | 0 | 1 | 1 | 0 | 1 | 1 |
| Park 2019 | 7 | 1 | 1 | 1 | 1 | 0 | 0 | 1 | 1 | 0 | 1 | 1 |
| Piron 2009 | 7 | 0 | 1 | 1 | 1 | 0 | 0 | 1 | 1 | 0 | 1 | 1 |
| Piron 2010 | 8 | 1 | 1 | 1 | 1 | 0 | 0 | 1 | 1 | 1 | 1 | 1 |
| Rogers | 6 | 1 | 1 | 1 | 1 | 0 | 0 | 0 | 1 | 0 | 1 | 1 |
| Prange 2015 | 7 | 1 | 1 | 1 | 1 | 0 | 0 | 1 | 1 | 0 | 1 | 1 |
| Schuster-Amft 2018 | 8 | 1 | 1 | 1 | 1 | 0 | 0 | 1 | 1 | 1 | 1 | 1 |
| Shin 2014 | 5 | 1 | 1 | 0 | 1 | 0 | 0 | 1 | 1 | 0 | 1 | 0 |
| Shin 2015 | 6 | 1 | 1 | 0 | 1 | 0 | 0 | 1 | 1 | 0 | 1 | 1 |
| Shin 2016 | 7 | 1 | 1 | 1 | 1 | 1 | 0 | 1 | 0 | 1 | 1 | 1 |
| Subramanian 2012 | 7 | 0 | 1 | 1 | 1 | 0 | 0 | 1 | 1 | 0 | 1 | 1 |
| Thielbar 2014 | 6 | 0 | 1 | 0 | 1 | 0 | 0 | 1 | 1 | 0 | 1 | 1 |
| Thielbar 2019 | 5 | 0 | 1 | 0 | 1 | 0 | 0 | 0 | 1 | 0 | 1 | 1 |
| Tomic 2017 | 7 | 1 | 1 | 0 | 1 | 0 | 0 | 1 | 1 | 1 | 1 | 1 |
| Wolf 2015 | 7 | 1 | 1 | 0 | 1 | 0 | 0 | 1 | 1 | 1 | 1 | 1 |
| Yin 2014 | 6 | 1 | 1 | 1 | 1 | 0 | 0 | 0 | 1 | 0 | 1 | 1 |
| Zondervan 2016 | 6 | 0 | 1 | 0 | 1 | 0 | 0 | 1 | 1 | 0 | 1 | 1 |

**Abbreviations:** Item 1: Eligibility criteria specified; Item 2: Random allocation; Item 3: Allocation concealment; Item 4: groups similar at baseline; Item 5: blinding of subjects; Item 6: blinding therapists; Item 7: blinding of assessors; Item 8: > 85% measures obtained; Item 9: "intention to treat" analysis; Item 10: between-group statistical comparisons are reported for at least one key outcome; Item 11: point measures and measures of variability for at least one key outcome

**Figure S1. Detailed analysis using the Cochrane Collaboration Risk of Bias Tool**

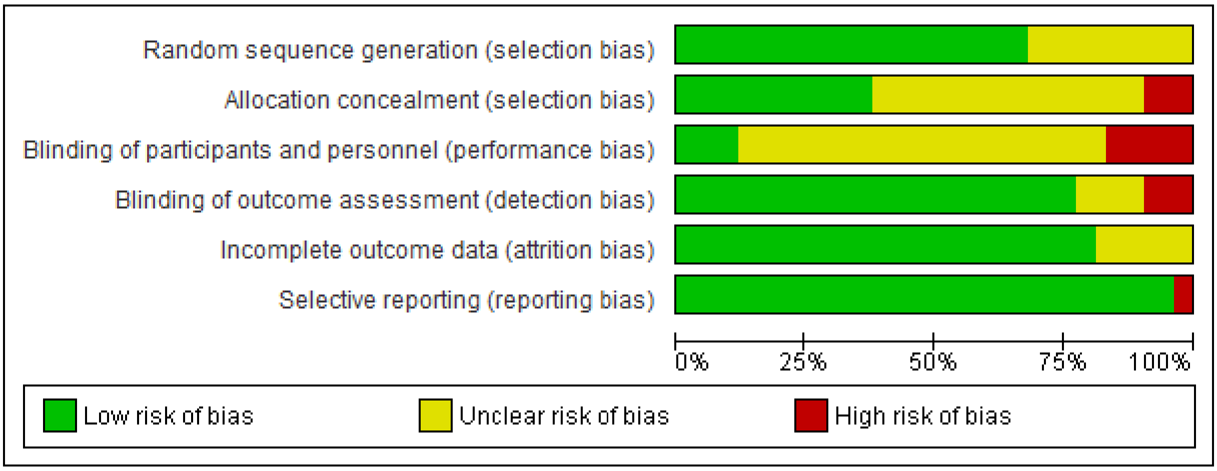

| | Random sequence generation (selection bias) | Allocation concealment (selection bias) | Blinding of participants and personnel (performance bias) | Blinding of outcome assessment (detection bias) | Incomplete outcome data (attrition bias) | Selective reporting (reporting bias) |
|---|---|---|---|---|---|---|
| Adomaviciene et al., 2019 | ? | − | + | + | + | + |
| Ang et al., 2014 | + | ? | − | + | + | + |
| Aprile et al., 2020 | + | − | ? | + | + | + |
| Askin et al., 2018 | + | − | − | + | + | + |
| Brunner et al., 2017 | + | + | − | + | + | + |
| Cameirao et al., 2011 | ? | ? | ? | + | + | + |
| Cameirao et al., 2012 | ? | ? | ? | + | + | + |
| Cho et al., 2019 | + | + | + | + | + | + |
| Choi et al., 2016 | ? | ? | + | + | + | + |
| Crosbie et al., 2012 | + | + | − | + | + | + |
| Dehem et al., 2019 | ? | ? | − | + | + | + |
| Duff et al., 2012 | ? | ? | ? | + | + | + |
| Faria et al., 2018 | + | ? | ? | − | ? | + |
| Henrique et al., 2019 | + | ? | ? | ? | + | + |
| Housman et al., 2009 | + | ? | ? | + | + | + |
| Hung et al., 2019 | + | + | − | + | + | + |
| Jang et al., 2005 | ? | ? | ? | ? | + | − |
| Jo et al., 2012 | + | ? | ? | ? | ? | + |
| Kim et al., 2013 | ? | ? | ? | ? | ? | + |
| Kim et al., 2018 | + | + | + | + | ? | + |
| Kiper et al., 2011 | ? | ? | ? | ? | + | + |
| Kiper et al., 2014 | ? | ? | ? | ? | ? | + |
| Kiper et al., 2018 | + | + | ? | − | ? | + |
| Klamroth-Marganska et al., 2014 | + | + | ? | + | + | + |
| Kottink et al., 2014 | ? | ? | ? | + | + | + |
| Kwon et al., 2012 | ? | ? | ? | + | + | + |
| Laffont et al., 2020 | + | + | ? | + | + | + |
| Lee et al., 2016 (a) | + | + | ? | + | + | + |
| Lee et al., 2016 (b) | + | ? | ? | + | + | + |
| Lee et al., 2018 | + | ? | ? | + | + | + |
| Levin et al., 2012 | + | ? | ? | + | + | + |
| Liao et al., 2012 | ? | + | ? | + | + | + |
| Mugler et al., 2019 | + | − | ? | + | + | + |
| Nijenhuis et al., 2016 | + | + | ? | − | + | + |
| Norousi-Gheidari et al., 2020 | + | − | − | + | + | + |
| Ogun et al., 2019 | + | ? | + | + | ? | + |
| Oh et al., 2019 | + | + | − | + | ? | + |
| Park et al., 2019 | + | + | ? | + | + | + |
| Piron et al., 2009 | + | + | ? | + | + | + |
| Piron et al., 2010 | + | + | ? | + | + | + |
| Prange et al., 2015 | + | + | − | + | + | + |
| Rogers et al., 2019 | + | + | ? | − | + | − |
| Schuster-Amft et al., 2018 | + | + | ? | + | + | + |
| Shin et al., 2014 | ? | ? | ? | + | + | + |
| Shin et al., 2015 | + | ? | ? | + | ? | + |
| Shin et al., 2016 | + | + | + | + | + | + |
| Subramanian et al., 2012 | + | ? | ? | + | + | + |
| Thielbar et al., 2014 | + | ? | ? | + | ? | + |
| Thielbar et al., 2019 | + | ? | ? | ? | + | + |
| Tomic et al., 2017 | ? | ? | ? | + | + | + |
| Wolf et al., 2015 | ? | ? | ? | + | + | + |
| Yin et al., 2014 | + | + | ? | − | + | + |
| Zondervan et al., 2016 | ? | ? | ? | + | + | + |

## Figure S2. Detailed summary of findings using the GRADEpro approach

**Author(s)**: Comparison of interventions using serious games to conventional therapies for upper limb rehabilitation after stroke
**Question**: Comparison of interventions using serious games to conventional therapies for upper limb rehabilitation after stroke
**Setting**:
**Bibliography**:

| Certainty assessment | | | | | | | № of patients | | Effect | | Certainty | Importance |
|---|---|---|---|---|---|---|---|---|---|---|---|---|
| № of studies | Study design | Risk of bias | Inconsistency | Indirectness | Imprecision | Other considerations | Intervention using serious game | Conventional therapy | Relative (95% CI) | Absolute (95% CI) | | |
| **Upper limb motor function (assessed with: FMA; Scale from: 0 to 66)** | | | | | | | | | | | | |
| 37 | randomised trials | not serious | very serious [a] | not serious | not serious | strong association [b,c] | 732 | 724 | - | SMD **0.47 SD higher** (0.24 higher to 0.7 higher) | ⊕⊕⊕◯ MODERATE | |
| **Upper limb motor function - number of Neurehabilitation principles > or = 8 (assessed with: FMA; Scale from: 0 to 66)** | | | | | | | | | | | | |
| 26 | randomised trials | not serious | very serious [a] | not serious | not serious | strong association [b,c] | 553 | 546 | - | SMD **0.62 SD higher** (0.33 higher to 0.92 higher) | ⊕⊕⊕◯ MODERATE | |
| **Upper limb motor function - number of Neurorehabilitation principles < 8 (assessed with: FMA; Scale from: 0 to 66)** | | | | | | | | | | | | |
| 11 | randomised trials | not serious | not serious | not serious | serious [d] | none [b,c] | 179 | 178 | - | SMD **0.05 SD higher** (0.17 lower to 0.27 higher) | ⊕⊕⊕◯ MODERATE | |
| **Activity (assessed with: ARAT, BBT, WMFT)** | | | | | | | | | | | | |
| 25 | randomised trials | not serious | serious [e] | not serious | not serious | none | 491 | 505 | - | SMD **0.25 SD higher** (0.05 higher to 0.46 higher) | ⊕⊕⊕◯ MODERATE | |
| **Activity - number of Neurehabilitation principles > or = 8 (assessed with: ARAT, BBT, WMFT)** | | | | | | | | | | | | |
| 17 | randomised trials | not serious | serious [e] | not serious | not serious | none | 310 | 316 | - | SMD **0.42 SD higher** (0.12 higher to 0.72 higher) | ⊕⊕⊕◯ MODERATE | |
| **Activity - number of Neurorehabilitation principles < 8 (assessed with: ARAT, BBT, WMFT)** | | | | | | | | | | | | |
| 8 | randomised trials | not serious | not serious | not serious | serious [d] | none | 181 | 189 | - | SMD **0.04 SD lower** (0.25 lower to 0.16 higher) | ⊕⊕⊕◯ MODERATE | |
| **Participation (assessed with: SIS (Social Participation Subscore))** | | | | | | | | | | | | |
| 4 | randomised trials | not serious | not serious | not serious | not serious | strong association | 60 | 60 | - | SMD **0.66 SD higher** (0.29 higher to 1.03 higher) | ⊕⊕⊕⊕ HIGH | |
| **Upper limb motor function - Subacute (assessed with: FMA; Scale from: 0 to 66)** | | | | | | | | | | | | |
| 13 | randomised trials | not serious | serious [e] | not serious | not serious | none | 360 | 365 | - | SMD **0.35 SD higher** (0.1 higher to 0.59 higher) | ⊕⊕⊕◯ MODERATE | |
| **Upper limb motor function - Chronic (assessed with: FMA; Scale from: 0 to 66)** | | | | | | | | | | | | |
| 23 | randomised trials | not serious | very serious [a] | not serious | not serious | strong association [b,c] | 360 | 347 | - | SMD **0.57 SD higher** (0.19 higher to 0.95 higher) | ⊕⊕⊕◯ MODERATE | |

**CI:** Confidence interval; **SMD:** Standardised mean difference

**Explanations**

a. The consistency was strongly downgraded because the heterogeneity was considerable (I2>75%)
b. Funnel plot did not show substantial asymmetry
c. Results were little affected by changes in methods or outliers through sensitivity analyses indicating robustness.
d. Precision was downgraded because confidence interval overlapped between serious game and control intervention
e. The consistency was downgraded because the heterogeneity was substantial (I2>30%)

**Figure S3.** Funnel Plot graphic representation

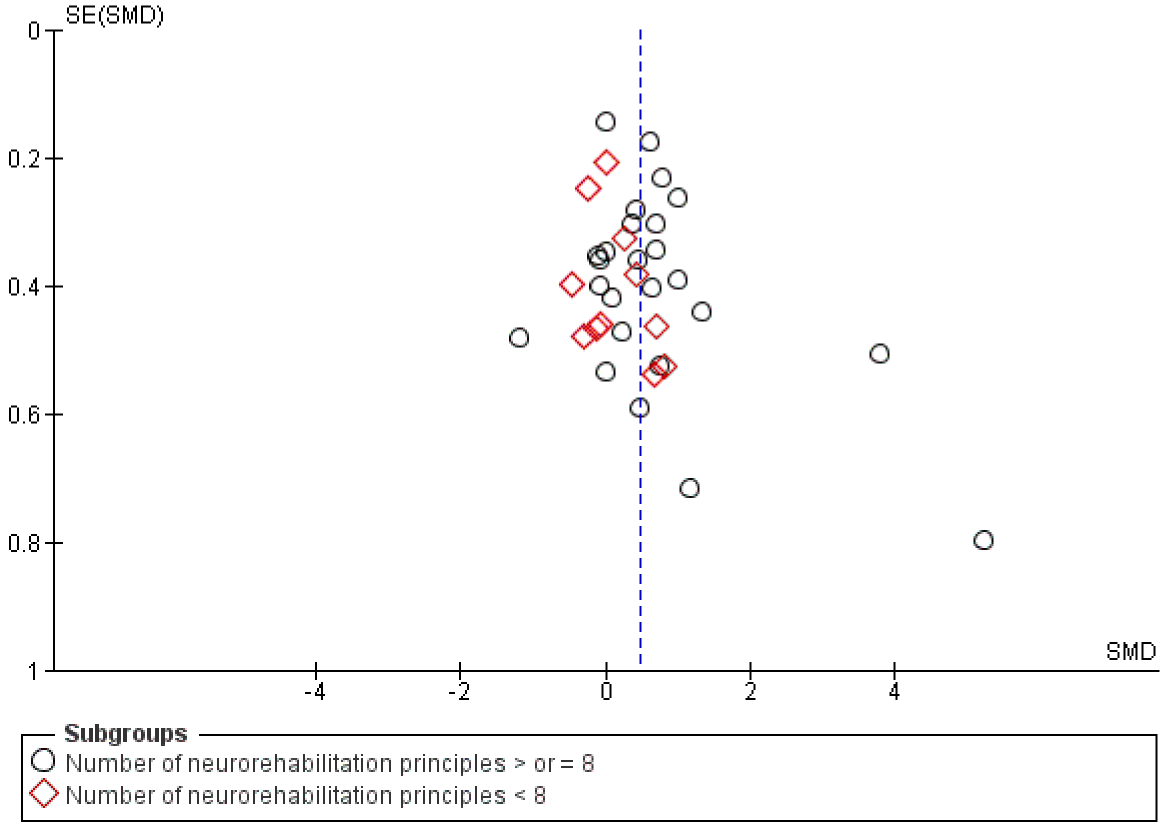

**Figure S4.** Sensitivity analysis without outliers. Forest plot of upper limb motor function as measured by the FMA-UE: studies using a serious game fulfilling ≥ 8 Npr versus studies using a serious game fulfilling < 8 Npr.

Abbreviations; FMA-UE, upper extremity subscale of the Fugl Meyer Assessment; Npr, Neurorehabilitation principles

| | Experimental | | | Control | | | | Std. Mean Difference |
|---|---|---|---|---|---|---|---|---|
| **Study or Subgroup** | **Mean** | **SD** | **Total** | **Mean** | **SD** | **Total** | **Weight** | **IV, Random, 95% CI** |
| **1.1.1 Number of neurorehabilitation principles > or = 8** | | | | | | | | |
| Aprile 2020 | 8.5 | 8.0668 | 91 | 8.57 | 8.0222 | 99 | 5.6% | -0.01 [-0.29, 0.28] |
| Choi 2016 | 19.08 | 13.39 | 12 | 9.67 | 14.22 | 12 | 2.4% | 0.66 [-0.17, 1.48] |
| Dehem 2019 | 19.5 | 18.55 | 15 | 10.8 | 19.58 | 17 | 3.0% | 0.44 [-0.26, 1.15] |
| Duff 2012 | 1.48 | 4.14 | 11 | 6.83 | 4.51 | 10 | 2.0% | -1.19 [-2.13, -0.24] |
| Henrique 2019 | 14.69 | 0.67 | 16 | 9.07 | 1.34 | 15 | 0.0% | 5.22 [3.66, 6.78] |
| Hung 2019 | 3.5 | 8.65 | 17 | 3.38 | 10.89 | 16 | 3.1% | 0.01 [-0.67, 0.69] |
| Jang 2005 | 7 | 4.33 | 5 | 2.4 | 2.69 | 5 | 1.1% | 1.15 [-0.25, 2.55] |
| Kiper 2011 | 9.8 | 10.32 | 40 | 1.6 | 10.91 | 40 | 4.4% | 0.76 [0.31, 1.22] |
| Kiper 2014 | 6.8 | 8.85 | 23 | 3.2 | 10.73 | 21 | 3.5% | 0.36 [-0.24, 0.96] |
| Kiper 2018 | 9.72 | 10.77 | 68 | 3.14 | 10.9 | 68 | 5.2% | 0.60 [0.26, 0.95] |
| Laffont 2019 | 24.1 | 14.8 | 25 | 17.8 | 14.6 | 26 | 3.8% | 0.42 [-0.13, 0.98] |
| Lee 2016 (a) | 7 | 10.99 | 13 | -0.31 | 11.51 | 13 | 2.6% | 0.63 [-0.16, 1.42] |
| Lee 2018 | 8.2 | 6.35 | 15 | 2.33 | 5.08 | 15 | 2.7% | 0.99 [0.23, 1.76] |
| Levin 2012 | 5 | 7.5 | 6 | 1 | 7.91 | 6 | 1.5% | 0.48 [-0.68, 1.63] |
| Norouzi-Gheidari 2019 | 2.1 | 11.42 | 9 | -0.2 | 7.54 | 9 | 2.1% | 0.23 [-0.70, 1.15] |
| Ogun 2019 | 6.91 | 5.37 | 33 | 1.5 | 5.44 | 32 | 4.0% | 0.99 [0.47, 1.51] |
| Oh 2019 | 1.9 | 3 | 17 | 2.1 | 2 | 14 | 2.9% | -0.07 [-0.78, 0.63] |
| Park 2019 | 1.9 | 2.8 | 12 | 2.1 | 2.7 | 13 | 2.6% | -0.07 [-0.86, 0.71] |
| Piron 2009 | 5.1 | 4.9 | 18 | 2.2 | 2.98 | 18 | 3.1% | 0.70 [0.02, 1.37] |
| Piron 2010 | 7.3 | 6.86 | 27 | 2.6 | 6.13 | 20 | 3.5% | 0.70 [0.11, 1.30] |
| Shin 2014 | 11.7 | 6.46 | 9 | 6.3 | 7.44 | 7 | 1.8% | 0.74 [-0.29, 1.77] |
| Shin 2015 | 4.63 | 6.48 | 16 | 5.57 | 10.25 | 16 | 3.0% | -0.11 [-0.80, 0.59] |
| Shin 2016 | 4.9 | 1 | 24 | 1.4 | 0.8 | 22 | 0.0% | 3.78 [2.79, 4.77] |
| Thielbar 2014 | 1.7 | 6.37 | 7 | 1.7 | 6.68 | 7 | 1.7% | 0.00 [-1.05, 1.05] |
| Tomic 2017 | 18 | 9.4 | 13 | 7.5 | 5.5 | 13 | 2.3% | 1.32 [0.46, 2.18] |
| Yin 2014 | 15.33 | 16.92 | 11 | 13.63 | 20.01 | 12 | 2.5% | 0.09 [-0.73, 0.91] |
| **Subtotal (95% CI)** | | | **513** | | | **509** | **70.4%** | **0.41 [0.22, 0.61]** |
| Heterogeneity: Tau² = 0.10; Chi² = 45.97, df = 23 (P = 0.003); I² = 50% | | | | | | | | |
| Test for overall effect: Z = 4.18 (P < 0.0001) | | | | | | | | |
| **1.1.2 Number of neurorehabilitation principles < 8** | | | | | | | | |
| Ang 2014 | 7.2 | 2.3 | 8 | 4.9 | 4.1 | 7 | 1.7% | 0.66 [-0.39, 1.72] |
| Askin 2018 | 2.25 | 6.3 | 18 | 0.5 | 7.25 | 20 | 3.3% | 0.25 [-0.39, 0.89] |
| Cameirao 2011 | 84.6 | 18.4 | 8 | 66.9 | 22.9 | 8 | 1.8% | 0.81 [-0.23, 1.84] |
| Housman 2009 | 3.3 | 2.4 | 14 | 2.2 | 2.6 | 14 | 2.7% | 0.43 [-0.32, 1.18] |
| Kim 2018 | 10.5 | 8.58 | 11 | 12 | 11.11 | 8 | 2.1% | -0.15 [-1.06, 0.76] |
| Kottink 2014 | 1.5 | 2.3 | 8 | 2.3 | 2.8 | 10 | 2.1% | -0.29 [-1.23, 0.64] |
| Kwon 2012 | 2.61 | 3.36 | 13 | 5.47 | 7.7 | 13 | 2.6% | -0.47 [-1.25, 0.31] |
| Liao 2012 | 6.3 | 5.64 | 10 | 1.3 | 7.92 | 10 | 2.1% | 0.70 [-0.21, 1.61] |
| Nijenhuis 2016 | 0.3 | 19.02 | 9 | 2 | 16.63 | 10 | 2.2% | -0.09 [-0.99, 0.81] |
| Prange 2015 | 7.9 | 8.3 | 33 | 10.2 | 9.6 | 33 | 4.2% | -0.25 [-0.74, 0.23] |
| Wolf 2015 | 9.3 | 25.76 | 47 | 9.6 | 24.98 | 45 | 4.7% | -0.01 [-0.42, 0.40] |
| **Subtotal (95% CI)** | | | **179** | | | **178** | **29.6%** | **0.05 [-0.17, 0.27]** |
| Heterogeneity: Tau² = 0.01; Chi² = 10.73, df = 10 (P = 0.38); I² = 7% | | | | | | | | |
| Test for overall effect: Z = 0.47 (P = 0.64) | | | | | | | | |
| **Total (95% CI)** | | | **692** | | | **687** | **100.0%** | **0.32 [0.16, 0.48]** |
| Heterogeneity: Tau² = 0.10; Chi² = 64.45, df = 34 (P = 0.001); I² = 47% | | | | | | | | |
| Test for overall effect: Z = 3.87 (P = 0.0001) | | | | | | | | |
| Test for subgroup differences: Chi² = 5.73, df = 1 (P = 0.02), I² = 82.6% | | | | | | | | |

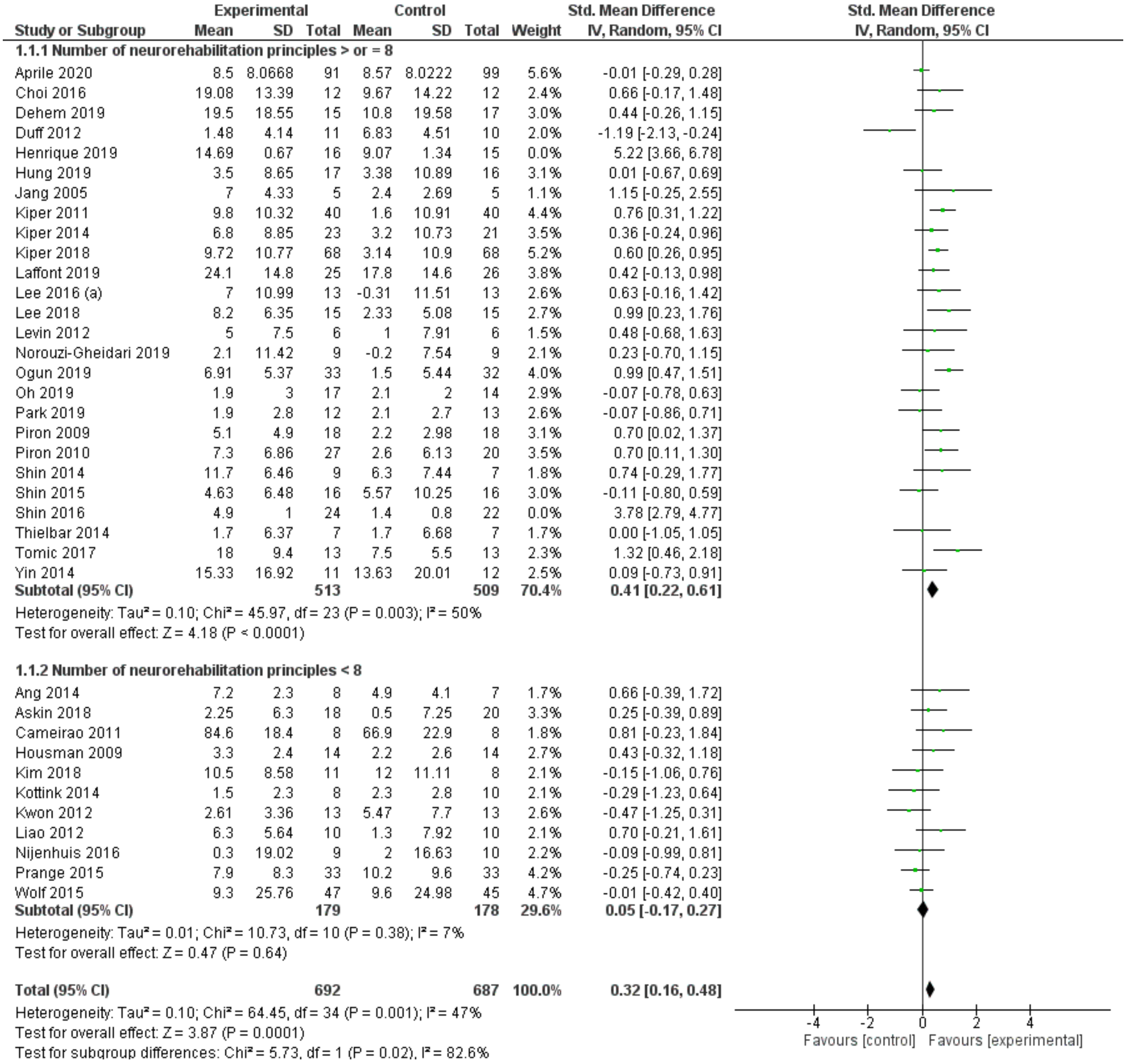

**Figure S5.** Sensitivity analysis: use of a different correlation coefficient value (0.9). Forest plot of upper limb motor function as measured by the FMA-UE: studies using a serious game fulfilling ≥ 8 Npr versus studies using a serious game fulfilling < 8 Npr.

Abbreviations; FMA-UE, upper extremity subscale of the Fugl Meyer Assessment; Npr, Neurorehabilitation principles

| | Experimental | | | Control | | | | Std. Mean Difference |
|---|---|---|---|---|---|---|---|---|
| Study or Subgroup | Mean | SD | Total | Mean | SD | Total | Weight | IV, Random, 95% CI |
| **1.2.1 Number of neurorehabilitation principles > or = 8** | | | | | | | | |
| Aprile 2020 | 8.5 | 8.0668 | 91 | 8.57 | 8.0222 | 99 | 3.5% | -0.01 [-0.29, 0.28] |
| Choi 2016 | 19.08 | 9.71 | 12 | 9.67 | 10.26 | 12 | 2.6% | 0.91 [0.06, 1.76] |
| Dehem 2019 | 19.5 | 13.68 | 15 | 10.8 | 14.4 | 17 | 2.9% | 0.60 [-0.11, 1.31] |
| Duff 2012 | 1.48 | 3.2 | 11 | 6.83 | 3.55 | 10 | 2.4% | -1.52 [-2.52, -0.53] |
| Henrique 2019 | 14.69 | 0.67 | 16 | 9.07 | 1.34 | 15 | 1.6% | 5.22 [3.66, 6.78] |
| Hung 2019 | 3.5 | 6.37 | 17 | 3.38 | 8.18 | 16 | 2.9% | 0.02 [-0.67, 0.70] |
| Jang 2005 | 7 | 3.12 | 5 | 2.4 | 1.96 | 5 | 1.6% | 1.59 [0.06, 3.13] |
| Kiper 2011 | 9.8 | 7.41 | 40 | 1.6 | 7.72 | 40 | 3.3% | 1.07 [0.60, 1.54] |
| Kiper 2014 | 6.8 | 6.45 | 23 | 3.2 | 7.64 | 21 | 3.1% | 0.50 [-0.10, 1.10] |
| Kiper 2018 | 9.72 | 7.74 | 68 | 3.14 | 7.76 | 68 | 3.4% | 0.84 [0.49, 1.20] |
| Laffont 2019 | 24.1 | 14.8 | 25 | 17.8 | 14.6 | 26 | 3.1% | 0.42 [-0.13, 0.98] |
| Lee 2016 (a) | 7 | 7.78 | 13 | -0.31 | 8.16 | 13 | 2.7% | 0.89 [0.08, 1.70] |
| Lee 2018 | 8.2 | 4.78 | 15 | 2.33 | 3.6 | 15 | 2.7% | 1.35 [0.55, 2.15] |
| Levin 2012 | 5 | 5.3 | 6 | 1 | 5.67 | 6 | 2.1% | 0.67 [-0.51, 1.85] |
| Norouzi-Gheidari 2019 | 2.1 | 8.2 | 9 | -0.2 | 5.37 | 9 | 2.5% | 0.32 [-0.62, 1.25] |
| Ogun 2019 | 6.91 | 3.85 | 33 | 1.5 | 3.86 | 32 | 3.2% | 1.39 [0.84, 1.93] |
| Oh 2019 | 1.9 | 3 | 17 | 2.1 | 2 | 14 | 2.9% | -0.07 [-0.78, 0.63] |
| Park 2019 | 1.9 | 2.8 | 12 | 2.1 | 2.7 | 13 | 2.7% | -0.07 [-0.86, 0.71] |
| Piron 2009 | 5.1 | 3.47 | 18 | 2.2 | 2.11 | 18 | 2.9% | 0.99 [0.29, 1.68] |
| Piron 2010 | 7.3 | 4.93 | 27 | 2.6 | 4.34 | 20 | 3.0% | 0.99 [0.37, 1.60] |
| Shin 2014 | 11.7 | 5.01 | 9 | 6.3 | 5.57 | 7 | 2.3% | 0.97 [-0.09, 2.03] |
| Shin 2015 | 4.63 | 4.7 | 16 | 5.57 | 7.25 | 16 | 2.9% | -0.15 [-0.84, 0.54] |
| Shin 2016 | 4.9 | 1 | 24 | 1.4 | 0.8 | 22 | 2.4% | 3.78 [2.79, 4.77] |
| Thielbar 2014 | 1.7 | 4.54 | 7 | 1.7 | 6.44 | 7 | 2.3% | 0.00 [-1.05, 1.05] |
| Tomic 2017 | 18 | 9.4 | 13 | 7.5 | 5.5 | 13 | 2.6% | 1.32 [0.46, 2.18] |
| Yin 2014 | 15.33 | 12.86 | 11 | 13.63 | 15.09 | 12 | 2.7% | 0.12 [-0.70, 0.94] |
| **Subtotal (95% CI)** | | | **553** | | | **546** | **70.2%** | **0.76 [0.43, 1.10]** |
| Heterogeneity: Tau² = 0.57; Chi² = 150.70, df = 25 (P < 0.00001); I² = 83% | | | | | | | | |
| Test for overall effect: Z = 4.53 (P < 0.00001) | | | | | | | | |
| **1.2.2 Number of neurorehabilitation principles < 8** | | | | | | | | |
| Ang 2014 | 7.2 | 2.3 | 8 | 4.9 | 4.1 | 7 | 2.3% | 0.66 [-0.39, 1.72] |
| Askin 2018 | 2.25 | 4.46 | 18 | 0.5 | 5.13 | 20 | 3.0% | 0.36 [-0.29, 1.00] |
| Cameirao 2011 | 84.6 | 18.4 | 8 | 66.9 | 22.9 | 8 | 2.3% | 0.81 [-0.23, 1.84] |
| Housman 2009 | 3.3 | 2.4 | 14 | 2.2 | 2.6 | 14 | 2.8% | 0.43 [-0.32, 1.18] |
| Kim 2018 | 10.5 | 6.34 | 11 | 12 | 7.86 | 8 | 2.5% | -0.20 [-1.12, 0.71] |
| Kottink 2014 | 1.5 | 2.3 | 8 | 2.3 | 2.8 | 10 | 2.5% | -0.29 [-1.23, 0.64] |
| Kwon 2012 | 3.36 | 2.75 | 13 | 5.47 | 7.07 | 13 | 2.8% | -0.38 [-1.16, 0.40] |
| Liao 2012 | 6.3 | 3.99 | 10 | 1.3 | 5.75 | 10 | 2.5% | 0.97 [0.03, 1.91] |
| Nijenhuis 2016 | 0.3 | 13.49 | 9 | 2 | 12.27 | 10 | 2.5% | -0.13 [-1.03, 0.78] |
| Prange 2015 | 7.9 | 8.3 | 33 | 10.2 | 9.6 | 33 | 3.3% | -0.25 [-0.74, 0.23] |
| Wolf 2015 | 9.3 | 19.34 | 47 | 9.6 | 18.86 | 45 | 3.4% | -0.02 [-0.42, 0.39] |
| **Subtotal (95% CI)** | | | **179** | | | **178** | **29.8%** | **0.09 [-0.15, 0.34]** |
| Heterogeneity: Tau² = 0.03; Chi² = 12.61, df = 10 (P = 0.25); I² = 21% | | | | | | | | |
| Test for overall effect: Z = 0.74 (P = 0.46) | | | | | | | | |
| **Total (95% CI)** | | | **732** | | | **724** | **100.0%** | **0.58 [0.32, 0.83]** |
| Heterogeneity: Tau² = 0.47; Chi² = 181.60, df = 36 (P < 0.00001); I² = 80% | | | | | | | | |
| Test for overall effect: Z = 4.39 (P < 0.0001) | | | | | | | | |
| Test for subgroup differences: Chi² = 10.15, df = 1 (P = 0.001), I² = 90.1% | | | | | | | | |

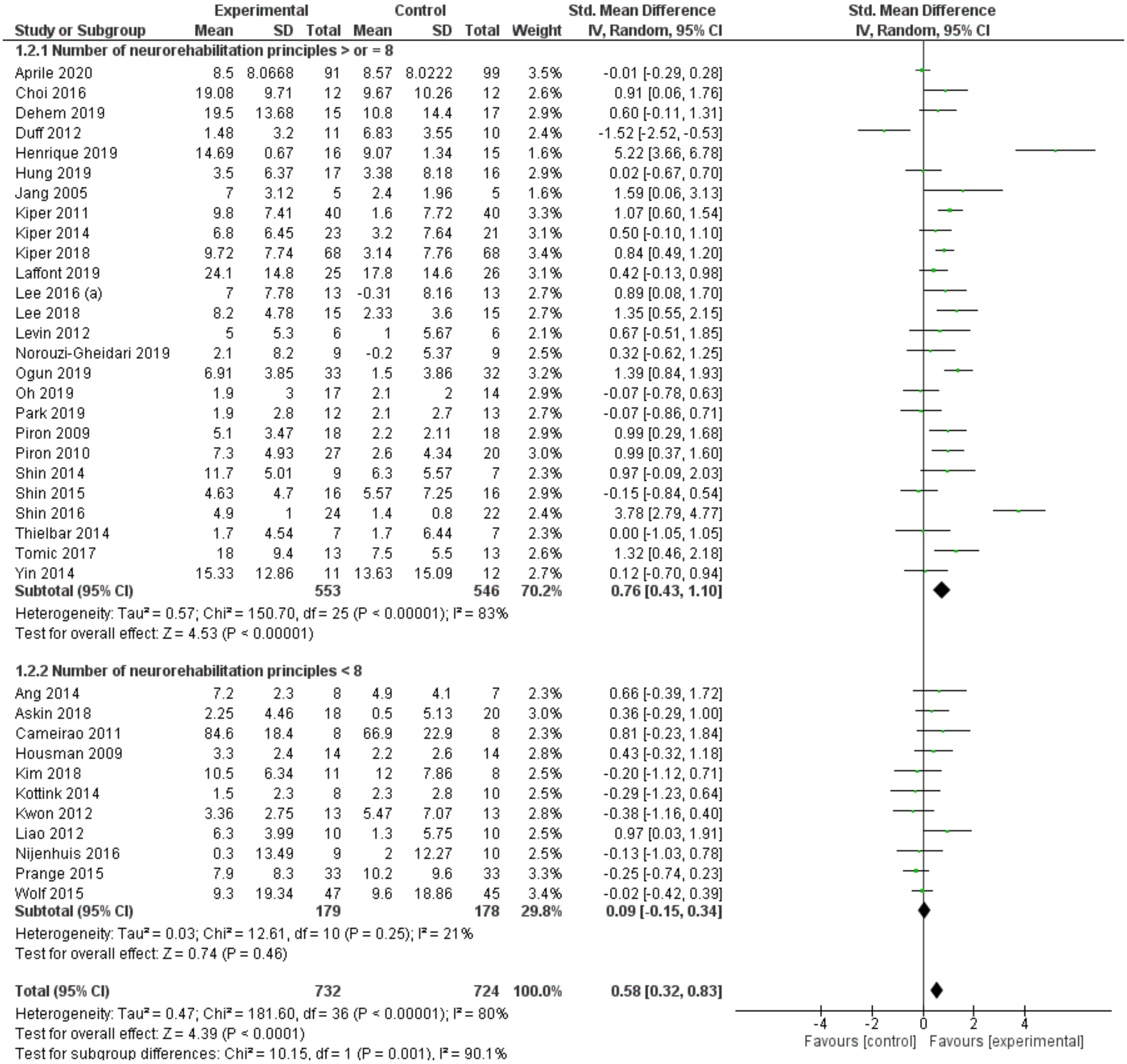

**Figure S6.** Forest plot of upper limb activity as measured by the ARAT, BBT, WMFT: studies in the subacute phase after stroke versus studies in the chronic phase after stroke

Abbreviations; ARAT, Action Research Arm Test; BBT, Box and Block test; WMFT, Wolf Motor Function Test; Npr, Neurorehabilitation principles

| | Experimental | | | Control | | | | Std. Mean Difference |
|---|---|---|---|---|---|---|---|---|
| **Study or Subgroup** | **Mean** | **SD** | **Total** | **Mean** | **SD** | **Total** | **Weight** | **IV, Random, 95% CI** |
| **2.3.1 Subacute** | | | | | | | | |
| Aprile 2020 | 6.04 | 8.547 | 91 | 6.7 | 9.3258 | 99 | 7.1% | -0.07 [-0.36, 0.21] |
| Brunner 2017 | 11.9 | 12.01 | 57 | 12.6 | 11.83 | 55 | 6.5% | -0.06 [-0.43, 0.31] |
| Dehem 2019 | 6.5 | 9.14 | 15 | 3.1 | 7.26 | 17 | 4.2% | 0.40 [-0.30, 1.11] |
| Kim 2018 | 8.4 | 8.68 | 11 | 9.6 | 10.35 | 8 | 3.2% | -0.12 [-1.03, 0.79] |
| Laffont 2019 | 15.7 | 16.3 | 25 | 7.4 | 12.2 | 26 | 5.1% | 0.57 [0.01, 1.13] |
| Rogers 2019 | 17.3 | 8.6 | 10 | 8.4 | 5.3 | 11 | 3.0% | 1.21 [0.26, 2.16] |
| Tomic 2017 | 14.1 | 7.9 | 13 | 6.7 | 7.8 | 13 | 3.6% | 0.91 [0.10, 1.73] |
| Yin 2014 | 18.34 | 19.99 | 11 | 17.02 | 21 | 12 | 3.6% | 0.06 [-0.76, 0.88] |
| **Subtotal (95% CI)** | | | **233** | | | **241** | **36.4%** | **0.27 [-0.04, 0.57]** |
| Heterogeneity: Tau² = 0.09; Chi² = 14.76, df = 7 (P = 0.04); I² = 53% | | | | | | | | |
| Test for overall effect: Z = 1.73 (P = 0.08) | | | | | | | | |
| **2.3.2 Chronic** | | | | | | | | |
| Askin 2018 | 1.75 | 13.3 | 18 | 0 | 7.81 | 20 | 4.6% | 0.16 [-0.48, 0.80] |
| Crosbie 2012 | 1.5 | 4.93 | 9 | 2.9 | 11.73 | 9 | 3.1% | -0.15 [-1.07, 0.78] |
| Duff 2012 | 0.47 | 0.36 | 11 | 0.54 | 0.28 | 10 | 3.4% | -0.21 [-1.07, 0.65] |
| Hung 2019 | 0.085 | 0.41 | 17 | 0.07 | 0.72 | 16 | 4.3% | 0.03 [-0.66, 0.71] |
| Jang 2005 | 4 | 4.84 | 5 | -1.6 | 5.91 | 5 | 1.8% | 0.94 [-0.41, 2.28] |
| Jo 2012 | 8.5 | 7.7 | 15 | 2 | 3.29 | 14 | 3.8% | 1.05 [0.27, 1.84] |
| Kottink 2014 | 3.9 | 4.9 | 8 | 5 | 5.1 | 10 | 3.1% | -0.21 [-1.14, 0.72] |
| Lee 2016 (a) | 2.85 | 10.47 | 13 | 2.54 | 10.48 | 13 | 3.9% | 0.03 [-0.74, 0.80] |
| Levin 2012 | 0.5 | 17.23 | 6 | 0.1 | 13.38 | 6 | 2.4% | 0.02 [-1.11, 1.16] |
| Nijenhuis 2016 | -0.37 | 21.77 | 9 | 3.23 | 15.24 | 10 | 3.2% | -0.18 [-1.09, 0.72] |
| Norouzi-Gheidari 2019 | 0.2 | 14.66 | 9 | -1.6 | 9.18 | 9 | 3.1% | 0.14 [-0.79, 1.07] |
| Ogun 2019 | 8.34 | 4.78 | 33 | 1.25 | 3.89 | 32 | 5.1% | 1.60 [1.04, 2.17] |
| Oh 2019 | 3.4 | 4.9 | 17 | 3.5 | 7.2 | 14 | 4.2% | -0.02 [-0.72, 0.69] |
| Park 2019 | 4.3 | 5.9 | 12 | 4.8 | 5.9 | 12 | 3.7% | -0.08 [-0.88, 0.72] |
| Schuster-Amft 2018 | 24.37 | 15.2 | 22 | 20.17 | 21.5 | 32 | 5.2% | 0.22 [-0.33, 0.76] |
| Thielbar 2014 | 3.1 | 5.6 | 7 | -3.5 | 8.01 | 7 | 2.4% | 0.89 [-0.22, 2.01] |
| Wolf 2015 | 5.1 | 22.85 | 47 | 8.8 | 23.19 | 45 | 6.2% | -0.16 [-0.57, 0.25] |
| **Subtotal (95% CI)** | | | **258** | | | **264** | **63.6%** | **0.23 [-0.05, 0.52]** |
| Heterogeneity: Tau² = 0.20; Chi² = 38.16, df = 16 (P = 0.001); I² = 58% | | | | | | | | |
| Test for overall effect: Z = 1.59 (P = 0.11) | | | | | | | | |
| **Total (95% CI)** | | | **491** | | | **505** | **100.0%** | **0.25 [0.05, 0.46]** |
| Heterogeneity: Tau² = 0.13; Chi² = 53.48, df = 24 (P = 0.0005); I² = 55% | | | | | | | | |
| Test for overall effect: Z = 2.41 (P = 0.02) | | | | | | | | |
| Test for subgroup differences: Chi² = 0.03, df = 1 (P = 0.87), I² = 0% | | | | | | | | |

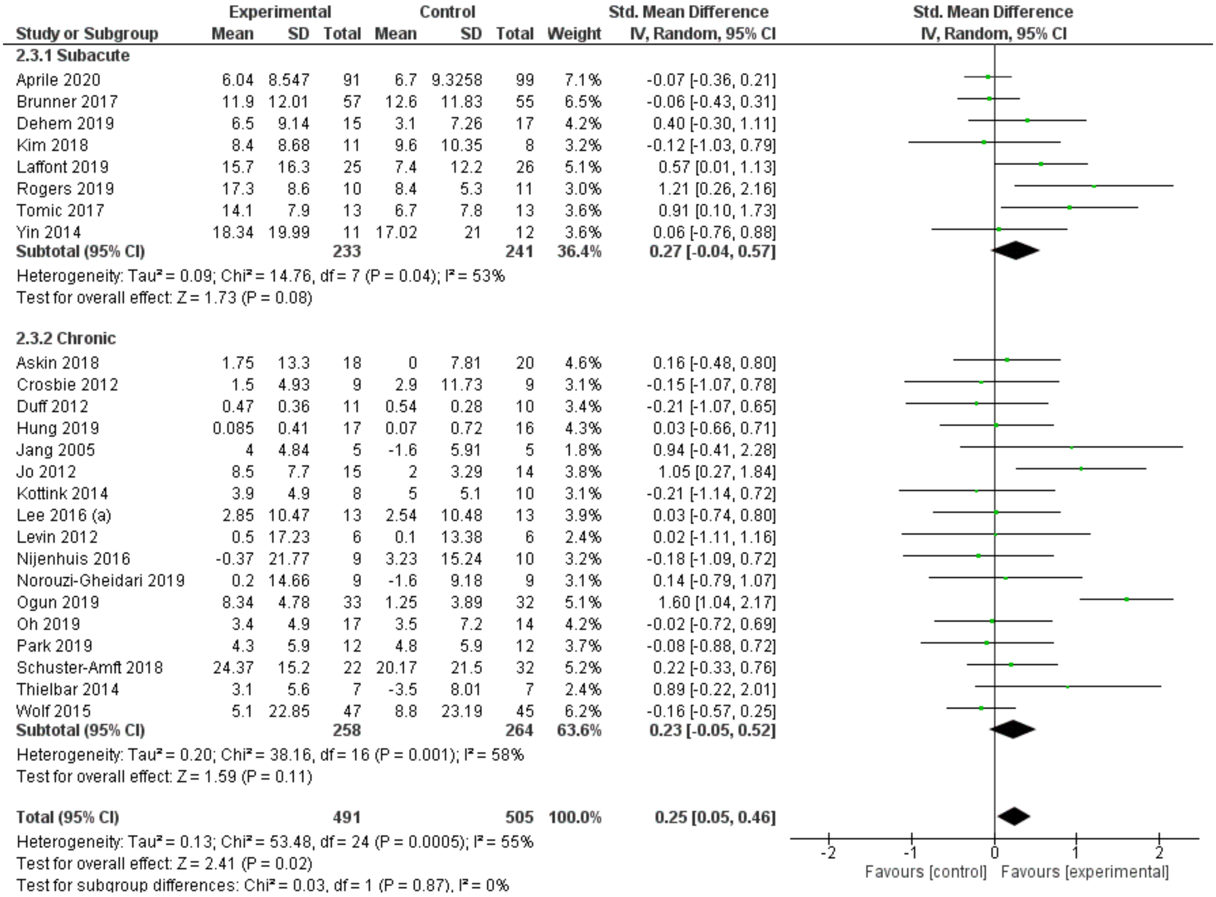

**Figure S7.** Follow-up evaluation. Forest plot of upper limb motor function as measured by the FMA-UE: studies using a serious game fulfilling ≥ 8 Npr versus studies using a serious game fulfilling < 8 Npr.

Abbreviations; FMA-UE, upper extremity subscale of the Fugl Meyer Assessment; Npr, Neurorehabilitation principles

| | Experimental | | | Control | | | | Std. Mean Difference |
|---|---|---|---|---|---|---|---|---|
| **Study or Subgroup** | **Mean** | **SD** | **Total** | **Mean** | **SD** | **Total** | **Weight** | **IV, Random, 95% CI** |
| **1.5.1 Number of neurorehabilitation principles > or = 8** | | | | | | | | |
| Aprile 2020 | 13.9 | 13.31 | 58 | 16.6 | 12.88 | 64 | 6.8% | -0.21 [-0.56, 0.15] |
| Choi 2016 | 29.25 | 13.91 | 12 | 13.17 | 14.88 | 12 | 5.2% | 1.08 [0.21, 1.94] |
| Dehem 2019 | 24.7 | 20.35 | 15 | 10 | 20.71 | 13 | 5.5% | 0.70 [-0.07, 1.46] |
| Hung 2019 | 3.5 | 9.58 | 17 | 4.48 | 11.98 | 16 | 5.8% | -0.09 [-0.77, 0.59] |
| Laffont 2019 | 31.2 | 15.8 | 23 | 25.6 | 16.3 | 23 | 6.2% | 0.34 [-0.24, 0.93] |
| Levin 2012 | 4 | 7.1 | 6 | 4.1 | 7.89 | 6 | 4.3% | -0.01 [-1.14, 1.12] |
| Norouzi-Gheidari 2019 | 3 | 11.29 | 9 | -0.4 | 8.02 | 9 | 5.0% | 0.33 [-0.60, 1.26] |
| Oh 2019 | 3.9 | 4.9 | 17 | 2.3 | 2.3 | 14 | 5.7% | 0.39 [-0.32, 1.11] |
| Park 2019 | 2.5 | 3.7 | 12 | 5.6 | 8.6 | 13 | 5.4% | -0.45 [-1.24, 0.35] |
| Piron 2009 | 4.6 | 4.8 | 18 | 1.5 | 3.1 | 18 | 5.8% | 0.75 [0.07, 1.43] |
| Shin 2016 | 5.3 | 1.1 | 24 | 1.3 | 0.8 | 22 | 4.6% | 4.06 [3.02, 5.10] |
| Thielbar 2014 | 1.3 | 5.85 | 7 | 3 | 5.79 | 7 | 4.6% | -0.27 [-1.33, 0.78] |
| Yin 2014 | 22.63 | 21.7 | 11 | 25.08 | 26.64 | 10 | 5.2% | -0.10 [-0.95, 0.76] |
| **Subtotal (95% CI)** | | | **229** | | | **227** | **70.3%** | **0.47 [-0.02, 0.96]** |
| Heterogeneity: Tau² = 0.65; Chi² = 70.33, df = 12 (P < 0.00001); I² = 83% | | | | | | | | |
| Test for overall effect: Z = 1.86 (P = 0.06) | | | | | | | | |
| **1.5.2 Number of neurorehabilitation principles < 8** | | | | | | | | |
| Ang 2014 | 9.7 | 2.9 | 8 | 3.6 | 5.9 | 7 | 4.3% | 1.27 [0.12, 2.41] |
| Cameirao 2011 | 79.1 | 19 | 8 | 72 | 18.8 | 8 | 4.8% | 0.36 [-0.64, 1.35] |
| Housman 2009 | 3.6 | 3.9 | 14 | 1.5 | 2.7 | 14 | 5.6% | 0.61 [-0.15, 1.37] |
| Kim 2018 | 17 | 7.08 | 11 | 18.1 | 10.9 | 8 | 5.0% | -0.12 [-1.03, 0.79] |
| Kottink 2014 | 4 | 5 | 8 | 4 | 3.2 | 10 | 5.0% | 0.00 [-0.93, 0.93] |
| Nijenhuis 2016 | 0.17 | 18.89 | 9 | 1.57 | 16.63 | 10 | 5.1% | -0.08 [-0.98, 0.83] |
| **Subtotal (95% CI)** | | | **58** | | | **57** | **29.7%** | **0.30 [-0.08, 0.69]** |
| Heterogeneity: Tau² = 0.01; Chi² = 5.26, df = 5 (P = 0.39); I² = 5% | | | | | | | | |
| Test for overall effect: Z = 1.54 (P = 0.12) | | | | | | | | |
| **Total (95% CI)** | | | **287** | | | **284** | **100.0%** | **0.42 [0.05, 0.79]** |
| Heterogeneity: Tau² = 0.49; Chi² = 75.61, df = 18 (P < 0.00001); I² = 76% | | | | | | | | |
| Test for overall effect: Z = 2.23 (P = 0.03) | | | | | | | | |
| Test for subgroup differences: Chi² = 0.27, df = 1 (P = 0.60), I² = 0% | | | | | | | | |

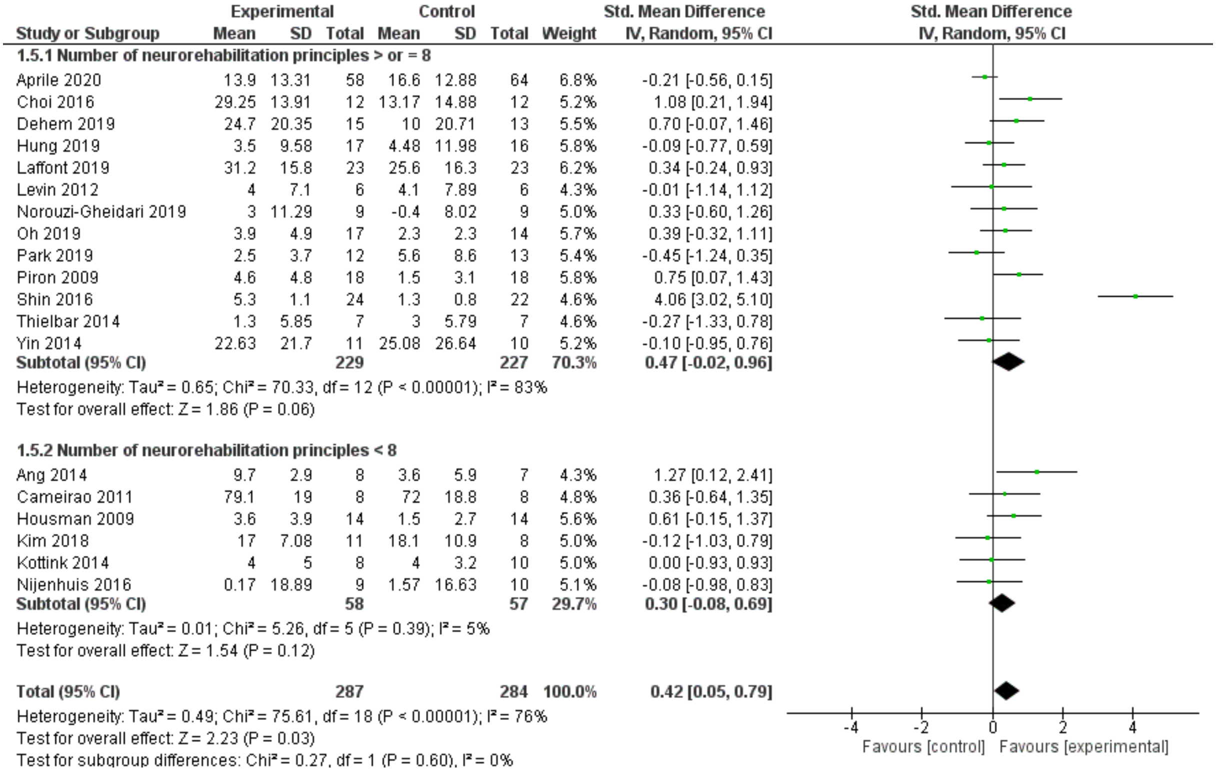

**Figure S8.** Follow-up evaluation. Forest plot of upper limb activity as measured by the ARAT, BBT, WMFT: studies using a serious game fulfilling ≥ 8 Npr versus studies using a serious game fulfilling < 8 Npr.

Abbreviations; ARAT, Action Research Arm Test; BBT, Box and Block test; WMFT, Wolf Motor Function Test; Npr, Neurorehabilitation principles

| | Experimental | | | Control | | | | Std. Mean Difference |
|---|---|---|---|---|---|---|---|---|
| **Study or Subgroup** | **Mean** | **SD** | **Total** | **Mean** | **SD** | **Total** | **Weight** | **IV, Random, 95% CI** |
| **2.2.1 Number of neurorehabilitation principles > or = 8** | | | | | | | | |
| Aprile 2020 | 11.6 | 12.37 | 58 | 13.8 | 11.96 | 64 | 13.5% | -0.18 [-0.54, 0.18] |
| Dehem 2019 | 9.7 | 11.77 | 15 | 1.3 | 5.89 | 13 | 5.0% | 0.86 [0.08, 1.64] |
| Hung 2019 | 0.015 | 0.37 | 17 | 0.08 | 0.72 | 16 | 6.2% | -0.11 [-0.80, 0.57] |
| Laffont 2019 | 23.8 | 16.7 | 23 | 14.4 | 15.5 | 23 | 7.6% | 0.57 [-0.02, 1.16] |
| Levin 2012 | 2.4 | 17.34 | 6 | 6.3 | 14.43 | 6 | 2.7% | -0.23 [-1.36, 0.91] |
| Norouzi-Gheidari 2019 | 1.5 | 13.23 | 9 | 2.7 | 10.45 | 10 | 4.0% | -0.10 [-1.00, 0.80] |
| Oh 2019 | 4.3 | 8.3 | 17 | 6.3 | 9.1 | 14 | 5.8% | -0.22 [-0.93, 0.49] |
| Park 2019 | 5.3 | 6.6 | 12 | 10.1 | 10.1 | 12 | 4.7% | -0.54 [-1.36, 0.27] |
| Rogers 2019 | 19 | 9.31 | 10 | 9.4 | 6.99 | 11 | 3.7% | 1.13 [0.19, 2.06] |
| Thielbar 2014 | 4.8 | 5.34 | 7 | -2.8 | 6.84 | 7 | 2.6% | 1.16 [-0.00, 2.32] |
| Yin 2014 | 28.97 | 23.62 | 11 | 34.17 | 16.17 | 10 | 4.3% | -0.24 [-1.10, 0.62] |
| **Subtotal (95% CI)** | | | **185** | | | **186** | **60.1%** | **0.14 [-0.18, 0.47]** |
| Heterogeneity: Tau² = 0.15; Chi² = 20.85, df = 10 (P = 0.02); I² = 52% | | | | | | | | |
| Test for overall effect: Z = 0.86 (P = 0.39) | | | | | | | | |
| **2.2.2 Number of neurorehabilitation principles < 8** | | | | | | | | |
| Brunner 2017 | 17.2 | 11.4 | 50 | 17.3 | 11.59 | 52 | 12.5% | -0.01 [-0.40, 0.38] |
| Crosbie 2012 | 0.8 | 5.1 | 9 | 3.4 | 11.76 | 9 | 3.8% | -0.27 [-1.20, 0.66] |
| Kim 2018 | 17.3 | 11.76 | 11 | 16.7 | 13.48 | 8 | 3.9% | 0.05 [-0.87, 0.96] |
| Kottink 2014 | 5.6 | 8.9 | 8 | 3.7 | 4.8 | 10 | 3.7% | 0.26 [-0.67, 1.20] |
| Nijenhuis 2016 | 0.33 | 21.77 | 9 | 2.03 | 13.45 | 10 | 4.0% | -0.09 [-0.99, 0.81] |
| Schuster-Amft 2018 | 24.77 | 17.63 | 22 | 21.7 | 21.5 | 32 | 8.5% | 0.15 [-0.39, 0.69] |
| Zondervan 2016 | 0.7 | 2.3 | 9 | -0.6 | 2.9 | 8 | 3.5% | 0.48 [-0.49, 1.44] |
| **Subtotal (95% CI)** | | | **118** | | | **129** | **39.9%** | **0.06 [-0.20, 0.31]** |
| Heterogeneity: Tau² = 0.00; Chi² = 1.71, df = 6 (P = 0.94); I² = 0% | | | | | | | | |
| Test for overall effect: Z = 0.44 (P = 0.66) | | | | | | | | |
| **Total (95% CI)** | | | **303** | | | **315** | **100.0%** | **0.09 [-0.10, 0.29]** |
| Heterogeneity: Tau² = 0.04; Chi² = 22.57, df = 17 (P = 0.16); I² = 25% | | | | | | | | |
| Test for overall effect: Z = 0.94 (P = 0.35) | | | | | | | | |
| Test for subgroup differences: Chi² = 0.17, df = 1 (P = 0.68), I² = 0% | | | | | | | | |

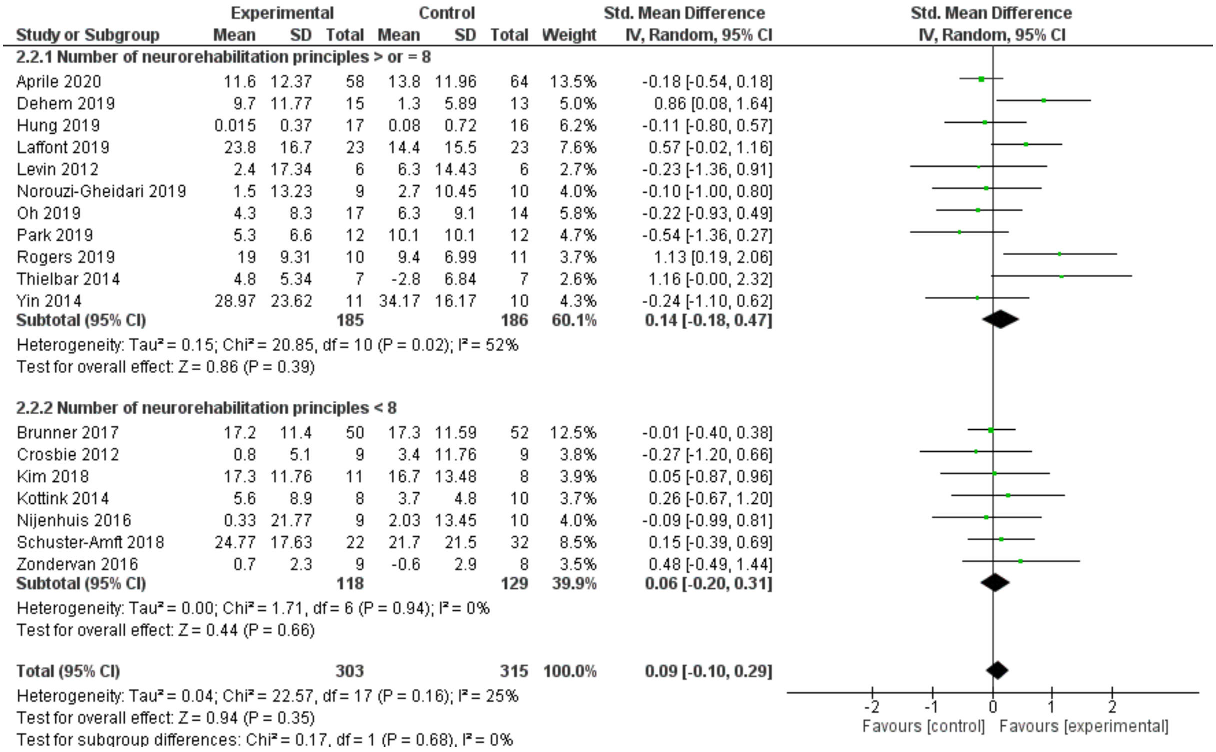

**Figure S9.** Follow-up evaluation. Forest plot of participation as measured by the social participation subscale of the SIS.

Abbreviations; SIS, Stroke Impact Scale

| | Experimental | | | Control | | | | Std. Mean Difference | Std. Mean Difference |
|---|---|---|---|---|---|---|---|---|---|
| **Study or Subgroup** | **Mean** | **SD** | **Total** | **Mean** | **SD** | **Total** | **Weight** | **IV, Random, 95% CI** | **IV, Random, 95% CI** |
| Dehem 2019 | 23.6 | 14.61 | 15 | 2.3 | 18.95 | 13 | 34.6% | 1.23 [0.41, 2.05] | |
| Norouzi-Gheidari 2019 | 6.9 | 16.45 | 9 | 3.8 | 11.09 | 9 | 30.1% | 0.21 [-0.72, 1.14] | |
| Park 2019 | 5.2 | 9.9 | 12 | 2.3 | 9.5 | 12 | 35.3% | 0.29 [-0.52, 1.09] | |
| **Total (95% CI)** | | | **36** | | | **34** | **100.0%** | **0.59 [-0.06, 1.25]** | |

Heterogeneity: Tau² = 0.15; Chi² = 3.55, df = 2 (P = 0.17); I² = 44%
Test for overall effect: Z = 1.78 (P = 0.08)

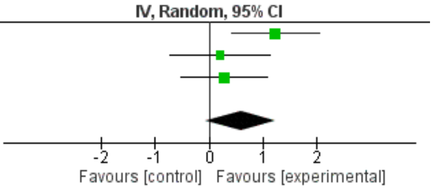

Supplement: Supplementary file 1 — Additional file 1: Table S1. Detailed PEDro scale scoring for each study. Figure S1. Detailed analysis using the Cochrane collaboration risk of bias tool. Figure S2. Detailed summary of findings using the GRADEpro approach. Figure S3. Funnel plot graphical representation. Figure S4. Sensitivity analysis without outliers. Figure S5. Sensitivity analysis: use of different correlation coefficient value (0.9). Forest plot of upper limb motor function as measured by the FMA-UE: studies using a serious game fulfilling ≥ 8 Npr versus studies using a serious game fulfilling < 8 Npr. Abbreviations; FMA-UE, upper extremity subscale of the Fugl Meyer Assessment; Npr, Neurorehabilitation principles. Figure S6. Forest plot of upper limb activity as measured by the ARAT, BBT, WMFT: studies in the subacute phase after stroke versus studies in the chronic phase after stroke. Abbreviations; ARAT, Action Research Arm Test; BBT, Box and Block test; WMFT, Wolf Motor Function Test; Npr, Neurorehabilitation principles. Figure S7. Follow-up evaluation. Forest plot of upper limb motor function as measured by the FMA-UE: studies using a serious game fulfilling ≥ 8 Npr versus studies using a serious game fulfilling < 8 Npr. Abbreviations; FMA-UE, upper extremity subscale of the Fugl Meyer Assessment; Npr, Neurorehabilitation principles. Figure S8. Follow-up evaluation. Forest plot of upper limb activity as measured by the ARAT, BBT, WMFT: studies using a serious game fulfilling ≥ 8 Npr versus studies using a serious game fulfilling < 8 Npr. Abbreviations; ARAT, Action Research Arm Test; BBT, Box and Block test; WMFT, Wolf Motor Function Test; Npr, Neurorehabilitation principles. Figure S9. Follow-up evaluation. Forest plot of participation as measured by the social participation subscale of the SIS. Abbreviations; SIS, Stroke Impact Scale. [file 12984_2021_889_MOESM1_ESM.pdf]
